# Supplementary material for: Genetic diversity, phylogenetic and phylogeographic analysis of Anopheles culicifacies species complex using ITS2 and COI sequences
Source: PLoS One. 2023 Aug 16;18(8):e0290178. doi: 10.1371/journal.pone.0290178 (PMC10431676; doi:10.1371/journal.pone.0290178)
Supplement: S1 Table — (PDF) [file pone.0290178.s001.pdf]

S1 Table. GenBank accession number, country of origin, sibling species of ITS2 sequences of *An. culicifacies.*

**No.**	**GenBank accession number**	**Country of origin**	**Sibling species**	**Sequence count for each country**
**1**	AY427755	India	-	21
**2**	AY427754	India	-	
**3**	EF192274	India	-	
**4**	AJ534645	India:Ramanathapuram	E	
**5**	AJ534644	India:Jabalpure	D	
**6**	AJ534643	India:Ladpur	C	
**7**	AJ534247	India:Ladpur	B	
**8**	AJ534246	India:Dehra	A	
**9**	EF462897	India	A	
**10**	EF462896	India	B	
**11**	EU882741	India: Rajasthan state, Alwar	D	
**12**	EU882740	India: Rajasthan state, Alwar	D	
**13**	EU882739	India: Rajasthan state, Alwar	D	

**14**	EU882738	India: Gujarat state, Surat	C	
**15**	EU882737	India: Gujarat state, Surat	C	
**16**	EU882736	India: Gujarat state, Surat	B	
**17**	EU882735	India: Gujarat state, Surat	B	
**18**	AF479315	India: Lahore	A	
**19**	AF479314	India: Delhi	B	
**20**	AF479313	India: Delhi	B	
**21**	MH187964	India	-	
**22**	AF402297	Iran	A	6
**23**	AY702488	Iran: Khash District, Sistan and Baluchistan	A	
**24**	AY702487	Iran: Sarbaz District, Sistan and Baluchistan	A	
**25**	JF966735	Iran	A	
**26**	JF966734	Iran	-	
**27**	AY702489	Iran: Khash District, Sistan and Baluchistan	A	
**28**	KY000682	Sri Lanka	-	3
**29**	AY167747	Sri Lanka	B	

**30**	AY168883	Sri Lanka	E	
**31**	AF479312	Cambodia: Rattanakiry	B	3
**32**	AF479311	Cambodia	B	
**33**	AF440396	Cambodia: Rattanakiry	B	
**34**	AY007172	China: Zhoujue County, Sichuan	-	2
**35**	AY007168	China: Zhoujue County, Sichuan	-	
