# Supplementary material for: Genetic diversity, phylogenetic and phylogeographic analysis of Anopheles culicifacies species complex using ITS2 and COI sequences
Source: PLoS One. 2023 Aug 16;18(8):e0290178. doi: 10.1371/journal.pone.0290178 (PMC10431676; doi:10.1371/journal.pone.0290178)
Supplement: S2 Table — (PDF) [file pone.0290178.s002.pdf]

S2 Table. GenBank accession number, country of origin, sibling species of *COI* sequences of *An. culicifacies*.

No.	GenBank accession number	Country of origin	Sibling species	Sequence count for each country
**1**	MH512896	India	-	38
**2**	MH330155	India	-	
**3**	MH507078	India	-	
**4**	MF893328	India: Dehriyan, Kangra	-	
**5**	DQ424962	India: Tamil Nadu	-	
**6**	GQ259185	India: Koraput, Orissa	-	
**7**	GQ259184	India: Koraput, Orissa	-	
**8**	GQ259183	India: Koraput, Orissa	-	
**9**	GQ259182	India: Koraput, Orissa	-	
**10**	LR736009	India:Tamilnadu	-	
**11**	LR736008	India:Tamilnadu	-	
**12**	LR736007	India:Tamilnadu	-	
**13**	AY917198	India: Jharkhand	-	
**14**	KR817729	India: Ooty	-	
**15**	EU143302	India	-	
**16**	EU143301	India	-	

**17**	EU143300	India	-	
**18**	AY834239	India	A	
**19**	AF117802	India:Delhi	B	
**20**	AF117801	India:Delhi	B	
**21**	AF117800	India:Delhi	B	
**22**	FJ424037	India	A	
**23**	FJ424038	India	A	
**24**	FJ424039	India	A	
**25**	FJ424040	India	A	
**26**	FJ424043	India	B	
**27**	FJ424044	India	B	
**28**	FJ424045	India	B	
**29**	FJ424046	India	B	
**30**	FJ424047	India	B	
**31**	FJ424048	India	B	
**32**	FJ424049	India	B	
**33**	FJ424052	India	C	
**34**	FJ424053	India	C	
**35**	FJ424054	India	C	
**36**	FJ424055	India	C	
**37**	FJ424056	India	D	
**38**	FJ424057	India	E	
**39**	KJ010896	Sri Lanka: Unnichchai	-	25

**40**	MH330212	Sri Lanka: Pasyala	-	
**41**	MH330211	Sri Lanka: Mirigama	-	
**42**	KX599421	Sri Lanka: Nuwara-Eliya	-	
**43**	KX599420	Sri Lanka: Jaffna	-	
**44**	KX599419	Sri Lanka: Nuwara-Eliya	-	
**45**	KX599418	Sri Lanka: Jaffna	-	
**46**	AF117798	Sri Lanka:south	B	
**47**	AF117796	Sri Lanka:north	B	
**48**	AF116834	Sri Lanka: northwest	B	
**49**	KJ010890	Sri Lanka: Hambantota	-	
**50**	KJ010891	Sri Lanka: Hambantota	-	
**51**	KJ010892	Sri Lanka: Hambantota	-	
**52**	KJ010893	Sri Lanka: Hambantota	-	
**53**	KJ010894	Sri Lanka: Hambantota	-	
**54**	KJ010895	Sri Lanka: Kallady	E	
**55**	KJ010897	Sri Lanka: Unnichchai	B	
**56**	KJ010898	Sri Lanka: Unnichchai	B	
**57**	KP197031	Sri Lanka	E	
**58**	KP197032	Sri Lanka	B	
**59**	KP197033	Sri Lanka	B	
**60**	KP197034	Sri Lanka	B	
**61**	KP197035	Sri Lanka	B	
**62**	KP197036	Sri Lanka	B	

**63**	AF117799	Sri Lanka:South	B	
**64**	KF406661	Pakistan: Khyber Pakhtunkhwa, Peshawar	A	8
**65**	KF406660	Pakistan: Khyber Pakhtunkhwa, Peshawar, Campus	-	
**66**	KF406659	Pakistan: Punjab, Sheikhupura, Mandi Safderabad	-	
**67**	KF406658	Pakistan: Punjab, Jehlum, Sohawa GT Road	-	
**68**	KF406657	Pakistan: Khyber Pakhtunkhwa, Peshawar	-	
**69**	KF406656	Pakistan: Punjab, Lahore, Lahore, Jump Wali Pulli	-	
**70**	AF117795	Pakistan:north	A	
**71**	AF117794	Pakistan:north	A	
**72**	AF116829	Iran: Zahedan	A	3
**73**	AF117793	Iran:Zahedan	A	
**74**	JF966744	Iran	A	
**75**	AF440397	North-eastern Cambodia at Char Ong Chan village, Ratanakiri	-	1

**76**	AF117797	Oman	A	1
**77**	MK170085	United Arab Emirates: Al Ain	-	1
