# Supplementary material for: Genetic diversity, phylogenetic and phylogeographic analysis of Anopheles culicifacies species complex using ITS2 and COI sequences
Source: PLoS One. 2023 Aug 16;18(8):e0290178. doi: 10.1371/journal.pone.0290178 (PMC10431676; doi:10.1371/journal.pone.0290178)
Supplement: S3 Table — (PDF) [file pone.0290178.s003.pdf]

S3 Table. GenBank accession numbers of ITS2 and *COI* sequences of the species used as outgroup of phylogenetic trees and phylogeographic trees.

**Species**	**ITS2**	***COI***
Ae. aegypti	AB548801	KC970274
Ae. aegypti	AB548800	KC970273
Ae. aegypti	AB548799	KC970272
Ae. aegypti	AB548798	KC970271
An. subpictus	KP165078	KC970282
An. subpictus	KP165079	KC970283
