# Supplementary material for: Genetic diversity, phylogenetic and phylogeographic analysis of Anopheles culicifacies species complex using ITS2 and COI sequences
Source: PLoS One. 2023 Aug 16;18(8):e0290178. doi: 10.1371/journal.pone.0290178 (PMC10431676; doi:10.1371/journal.pone.0290178)
Supplement: S4 Table — (PDF) [file pone.0290178.s004.pdf]

S4 Table. GenBank accession numbers of the sequences included in clade 1, 2 and outgroup of Bayesian likelihood tree generated by MrBayes-3.2.5_WIN32_x86 software using ITS2 sequences of *An. culicifacies*.

**Clade 1**	**Clade 2**	**Outgroup**
AF440396	AF402297	AB548798
AF479311	AF479315	AB548801
AF479312	AJ534246	AB548799
AF479313	AJ534644	AB548800
AF479314	AY427755	
AJ534247	AY702487	
AJ534643	AY702488	
AJ534645	AY702489	
AY007168	EF462897	
AY007172	EU882739	
AY167747	EU882740	
AY168883	EU882741	
AY427754	JF966734	
EF192274	JF966735	
EF462896		
EU882735		
EU882736		
EU882737		

EU882738		
KY000682		
MH187964		
