# Supplementary material for: Genetic diversity, phylogenetic and phylogeographic analysis of Anopheles culicifacies species complex using ITS2 and COI sequences
Source: PLoS One. 2023 Aug 16;18(8):e0290178. doi: 10.1371/journal.pone.0290178 (PMC10431676; doi:10.1371/journal.pone.0290178)
Supplement: S5 Table — (PDF) [file pone.0290178.s005.pdf]

S5 Table. GenBank accession numbers of the sequences included in Clade 1 to 4 and outgroup of Bayesian likelihood tree generated by MrBayes-3.2.5_WIN32_x86 software using *COI* sequences of *An. culicifacies*.

**Clade 1**	**Clade 2**	**Clade 3**	**Clade 4**	**Outgroup**
DQ424962	AF116834	AF117793	AY834239	KC970271
EU143300	AF117796	AF117794	KF406656	KC970272
EU143302	AF117798	FJ424037	KF406658	KC970273
GQ259182	AF117799	FJ424038	KF406659	KC970274
GQ259185	AF117800	FJ424039	KF406660	
KJ010896	AF117801	FJ424040	KR817729	
KX599420	FJ424053	FJ424056	MK170085	
MF893328	FJ424054	AF116829	KF406657	
MH330155	FJ424055	AF117795		
MH330211	AF117802	AF117797		
KJ010895	FJ424045			
GQ259184	AF440397			
KJ010897	FJ424043			
KJ010898	FJ424044			
KP197032	FJ424046			
KF406661	FJ424047			
KJ010890	FJ424048			
KJ010891	FJ424049			

KJ010892	FJ424052			
KJ010893	FJ424057			
KJ010894				
KP197031				
KP197033				
KP197034				
KP197035				
KP197036				
KX599418				
KX599419				
KX599421				
LR736007				
LR736008				
MH330212				
MH507078				
MH512896				
