# Supplementary material for: Genetic diversity, phylogenetic and phylogeographic analysis of Anopheles culicifacies species complex using ITS2 and COI sequences
Source: PLoS One. 2023 Aug 16;18(8):e0290178. doi: 10.1371/journal.pone.0290178 (PMC10431676; doi:10.1371/journal.pone.0290178)
Supplement: S6 Table — (PDF) [file pone.0290178.s006.pdf]

S6 Table. Haplotype frequencies and haplotype diversities (Hd) in populations of ITS2 haplotype network of *An. culicifacies* species complex obtained using Arlequin version 3.5.2.2 and DnaSP v5 software.

**Haplotype**	**Iran (6)**	**Cambodia (3)**	**India (21)**	**China (2)**	**Sri Lanka (3)**
**Hap_1**	1	0	0	0	0
**Hap_2**	0	3	4	0	0
**Hap_3**	0	0	1	0	0
**Hap_4**	0	0	3	0	0
**Hap_5**	0	0	1	0	0
**Hap_6**	0	0	1	0	0
**Hap_7**	0	0	1	0	0
**Hap_8**	0	0	1	0	0
**Hap_9**	0	0	0	1	0
**Hap_10**	0	0	0	1	0
**Hap_11**	0	0	1	0	3
**Hap_12**	0	0	1	0	0
**Hap_13**	0	0	1	0	0
**Hap_14**	4	0	1	0	0
**Hap_15**	0	0	1	0	0
**Hap_16**	0	0	1	0	0
**Hap_17**	0	0	1	0	0
**Hap_18**	0	0	1	0	0

**Hap_19**	1	0	0	0	0
**Hap_20**	0	0	1	0	0
**Haplotype diversity (Hd)**	0.333	0.000	0.729	0.000	0.000
