# Supplementary material for: Genetic diversity, phylogenetic and phylogeographic analysis of Anopheles culicifacies species complex using ITS2 and COI sequences
Source: PLoS One. 2023 Aug 16;18(8):e0290178. doi: 10.1371/journal.pone.0290178 (PMC10431676; doi:10.1371/journal.pone.0290178)
Supplement: S7 Table — (PDF) [file pone.0290178.s007.pdf]

S7 Table. Haplotype frequencies and haplotype diversities (Hd) in populations of *COI* haplotype network of *An. culicifacies* species complex obtained using Arlequin version 3.5.2.2 and DnaSP v5 software.

**Haplotype**	**Iran (3)**	**Sri Lanka (25)**	**Pakistan (8)**	**Oman (1)**	**India (38)**	**Cambodia (1)**	**United Arab Emirates (1)**
**Hap_1**	1	0	0	0	0	0	0
**Hap_2**	0	2	0	0	0	0	0
**Hap_3**	1	0	0	0	0	0	0
**Hap_4**	0	0	1	0	2	0	0
**Hap_5**	0	0	1	0	0	0	0
**Hap_6**	0	0	0	1	0	0	0
**Hap_7**	0	1	0	0	0	0	0
**Hap_8**	0	1	0	0	0	0	0
**Hap_9**	0	0	0	0	3	0	0
**Hap_10**	0	0	0	0	1	0	0
**Hap_11**	0	0	0	0	2	0	0
**Hap_12**	0	0	0	0	0	1	0
**Hap_13**	0	0	2	0	1	0	0
**Hap_14**	0	0	0	0	1	0	0
**Hap_15**	0	0	0	0	1	0	0
**Hap_16**	0	4	0	0	1	0	0
**Hap_17**	0	0	0	0	1	0	0

**Hap_18**	0	0	0	0	1	0	0
**Hap_19**	0	0	0	0	2	0	0
**Hap_20**	0	0	0	0	1	0	0
**Hap_21**	0	0	0	0	1	0	0
**Hap_22**	0	0	0	0	1	0	0
**Hap_23**	0	0	0	0	1	0	0
**Hap_24**	0	0	0	0	1	0	0
**Hap_25**	0	0	0	0	1	0	0
**Hap_26**	0	0	0	0	2	0	0
**Hap_27**	0	0	0	0	1	0	0
**Hap_28**	0	0	0	0	1	0	0
**Hap_29**	0	0	0	0	1	0	0
**Hap_30**	0	0	0	0	1	0	0
**Hap_31**	0	0	0	0	1	0	0
**Hap_32**	0	2	0	0	3	0	0
**Hap_33**	1	0	0	0	0	0	0
**Hap_34**	0	0	1	0	0	0	0
**Hap_35**	0	0	1	0	0	0	0
**Hap_36**	0	0	1	0	0	0	0
**Hap_37**	0	0	1	0	0	0	0
**Hap_38**	0	3	0	0	0	0	0
**Hap_39**	0	1	0	0	0	0	0
**Hap_40**	0	2	0	0	0	0	0

**Hap_41**	0	1	0	0	0	0	0
**Hap_42**	0	2	0	0	0	0	0
**Hap_43**	0	1	0	0	0	0	0
**Hap_44**	0	1	0	0	0	0	0
**Hap_45**	0	1	0	0	0	0	0
**Hap_46**	0	1	0	0	0	0	0
**Hap_47**	0	0	0	0	1	0	0
**Hap_48**	0	1	0	0	0	0	0
**Hap_49**	0	0	0	0	1	0	0
**Hap_50**	0	0	0	0	1	0	0
**Hap_51**	0	0	0	0	1	0	0
**Hap_52**	0	1	0	0	0	0	0
**Hap_53**	0	0	0	0	0	0	0
**Hap_54**	0	0	0	0	1	0	0
**Hap_55**	0	0	0	0	1	0	1
**Haplotype diversity (Hd)**	1.000	0.957	0.964	-	0.980	-	-
