# Supplementary material for: Genetic diversity, phylogenetic and phylogeographic analysis of Anopheles culicifacies species complex using ITS2 and COI sequences
Source: PLoS One. 2023 Aug 16;18(8):e0290178. doi: 10.1371/journal.pone.0290178 (PMC10431676; doi:10.1371/journal.pone.0290178)
Supplement: S8 Table — (PDF) [file pone.0290178.s008.pdf]

S8 Table. GenBank accession numbers of ITS2 sequences included in clade 1, 2 and outgroup of phylogeographic tree generated by BEAST v1.8.2 software using ITS2 sequences of *An. culicifacies.*

**Clade 1**	**Clade 2**	**Outgroup**
AJ534644	MH187964	KP165078
EF462897	AF440396	KP165079
AY702487	AY167747	
EU882741	AF479313	
AY427755	EU882737	
EU882740	AF479314	
AF402297	EF192274	
AF479315	EF462896	
AY702489	AJ534643	
JF966734	KY000682	
EU882739	AJ534645	
AJ534246	AY168883	
AY702488	EU882738	
	AY007172	
	EU882735	
	EU882736	
	AF479311	
	AJ534247	

	AY427754	
	AF479312	
	AY007168	
