# Supplementary material for: Genetic diversity, phylogenetic and phylogeographic analysis of Anopheles culicifacies species complex using ITS2 and COI sequences
Source: PLoS One. 2023 Aug 16;18(8):e0290178. doi: 10.1371/journal.pone.0290178 (PMC10431676; doi:10.1371/journal.pone.0290178)
Supplement: S9 Table — (PDF) [file pone.0290178.s009.pdf]

**S9 Table.** Posterior probability values, Mean values of diversification times and 95% highest posterior density (HPD) of each nodes of the ITS2 phylogeographic tree.

| **Node** | **PP** | **Mean (Mya)** | **95% HPD (Mya)** |
|---|---|---|---|
| **A** | 1 | 83.06 | 81.15-85.09 |
| **B** | 1 | 22.19 | 20.25-24.12 |
| **C** | 0.963 | 16.25 | 9.77-22.12 |
| **D** | 0.217 | 19.17 | 13.62-23.34 |
| **E** | 0 | 12.11 | 10.17-12.98 |
| **F** | 0 | 10.85 | 7.70-14.71 |
| **G** | 0.003 | 8.73 | 1.94-18.23 |
| **H** | 0 | 16.47 | 19.17-13.62 |
| **I** | 0 | 16.18 | 17.55-13.63 |
| **J** | 0.005 | 5.53 | 0.60-12.07 |
| **K** | 0.003 | 6.83 | 1.38-16.57 |
| **L** | 0.034 | 3.84 | 0.06-10.44 |
| **M** | 0.007 | 5.80 | 0.12-11.73 |
| **N** | 0.003 | 5.80 | 0.64-13.48 |
