# Supplementary material for: Genetic diversity, phylogenetic and phylogeographic analysis of Anopheles culicifacies species complex using ITS2 and COI sequences
Source: PLoS One. 2023 Aug 16;18(8):e0290178. doi: 10.1371/journal.pone.0290178 (PMC10431676; doi:10.1371/journal.pone.0290178)
Supplement: S10 Table — (PDF) [file pone.0290178.s010.pdf]

S10 Table. GenBank accession numbers of *COI* sequences included in clade 1 to 3 and outgroup of phylogeographic tree generated by BEAST v1.8.2 software using *COI* sequences of *An. culicifacies.*

**Clade 1**	**Clade 2**	**Clade 3**	**Outgroup**
AF117793	KJ010892	JF966744	KC970283
FJ424037	MH512896	MH507078	KC970282
FJ424038	KJ010898	KX599419	
AF117794	KJ010897	KX599418	
AF117795	GQ259184	KP197036	
AF116829	KP197032	EU143300	
AF117797	KP197034	KJ010890	
FJ424039	LR736008	KF406660	
FJ424040	MH330212	KF406657	
FJ424056	KP197033	AY834239	
FJ424043	GQ259183	KF406656	
AF117796	KJ010894	KF406658	
AF116834	AY917198	KR817729	
FJ424052	EU143302	MK170085	
AF117799	DQ424962	KF406659	
AF117798	EU143301	LR736009	
FJ424057	KP197035	KX599421	
FJ424049	KJ010891		

AF440397	KJ010893		
AF117800	KP197031		
FJ424044	KF406661		
FJ424047	LR736007		
FJ424048	KX599420		
FJ424053	MH330155		
AF117801	GQ259182		
FJ424054	KJ010895		
FJ424055			
AF117802			
FJ424045			
FJ424046			
