# Supplementary material for: Genetic diversity, phylogenetic and phylogeographic analysis of Anopheles culicifacies species complex using ITS2 and COI sequences
Source: PLoS One. 2023 Aug 16;18(8):e0290178. doi: 10.1371/journal.pone.0290178 (PMC10431676; doi:10.1371/journal.pone.0290178)
Supplement: S11 Table — (PDF) [file pone.0290178.s011.pdf]

S11 Table. Posterior probability values, Mean values of diversification times and 95% highest posterior density (HPD) of each nodes of the *COI* phylogeographic tree.

Node	**PP**	**Mean (Mya)**	**95% HPD (Mya)**
A	1	82.75	80.93-85.07
B	0.98	24.33	22.37-26.22
C	1	17.30	10.36-24.68
D	0.04	18.07	13.24-23.46
E	0.02	17.92	13.17-24.28
F	0.09	10.11	4.02-17.12
G	0.33	9.59	3.19-16.05
H	0.50	11.77	5.79-17.9
I	0.40	12.18	5.45-19.96
J	0.01	16.35	10.37-21.15
K	0.31	7.22	1.40-14.14
L	0.07	6.18	0.74-12.70
M	0.51	3.28	0.11-7.36
N	0.97	8.39	2.83-14.72
