# Supplementary material for: Genetic diversity, phylogenetic and phylogeographic analysis of Anopheles culicifacies species complex using ITS2 and COI sequences
Source: PLoS One. 2023 Aug 16;18(8):e0290178. doi: 10.1371/journal.pone.0290178 (PMC10431676; doi:10.1371/journal.pone.0290178)
Supplement: Attachment — Submitted filename: PONE-D-23-08529.pdf [file pone.0290178.s013.pdf]

PLOS ONE

Genetic diversity, phylogenetic and phylogeographic analysis of Anopheles culicifacies species complex using ITS2 and COI sequences

--Manuscript Draft--

**Manuscript Number:**	PONE-D-23-08529
**Article Type:**	Research Article
**Full Title:**	Genetic diversity, phylogenetic and phylogeographic analysis of Anopheles culicifacies species complex using ITS2 and COI sequences
**Short Title:**	Phylogenetic and phylogeographic analysis of Anopheles culicifacies species complex
**Corresponding Author:**	B.G.D.N.K. De Silva, Ph.D<br>University of Sri Jayewardenepura<br>Nugegoda, SRI LANKA
**Keywords:**	Anopheles culicifacies, ITS2, COI, Phylogeny, Phylogeography
**Abstract:**	Anopheles culicifacies is the major vector of malaria in Sri Lanka and the Indian subcontinent which is characterized as a species complex with five sibling species provisionally designated as A, B, C, D and E. The current study was carried out to understand the phylogenetic and phylogeographic relationships between the sibling species of the species complex while observing their genetic diversity and genetic differentiation. The available ITS2 and COI sequences of An. culicifacies species complex reported from different geographical locations at the NCBI public database were used for the analysis. Bayesian likelihood trees were generated for the phylogenetic analysis. The divergence of the species complex was obtained from the Bayesian phylogeographic model in BEAST. There were two clades of the sibling species of An. culicifacies species complex as A, D and B, C and E in both phylogenetic and phylogeographic analysis using ITS2 sequences. Based on the highly divergent COI sequences and the high mutation rate of the mitochondrial genome, there were many clades in both phylogenetic and phylogeographic analysis using COI sequences. The diversification of An. culicifacies species complex was obtained as ranging from 20.25 to 24.12 Mya and 22.37 to 26.22 Mya based on ITS2 and COI phylogeographic analysis respectively. There was a recent diversification of the sibling species A and D than the sibling species B, C and E. Low haplotype diversity was observed in the sequences reported from Sri Lanka in both ITS2 and COI analysis that can be due to bottlenecks resulting from the intense malaria control efforts. A high genetic differentiation was achieved for some populations due to the large geographical distance. A high genetic diversity of An. culicifacies species complex was suggested through the obtained results implying the possibility of maintaining a relatively high effective population size despite the vector control efforts.
**Order of Authors:**	B.G.D.N.K. De Silva, Ph.D
	Rathnayake Achchillage Samanthika Rathnayake, B.Sc. special degree
	Methsala Wedage, B.Sc. special degree
	Lihini Muthukumarana, B.Sc. special degree
**Additional Information:**	
**Question**	**Response**
**Financial Disclosure**<br><br>Enter a financial disclosure statement that describes the sources of funding for the work included in this submission. Review the submission guidelines for detailed requirements. View published research articles from *PLOS ONE* for specific	The author(s) received no specific funding for this work.

Powered by Editorial Manager® and ProduXion Manager® from Aries Systems Corporation

examples.<br><br>This statement is required for submission and **will appear in the published article** if the submission is accepted. Please make sure it is accurate.<br><br>**Unfunded studies**<br>Enter: *The author(s) received no specific funding for this work.*<br><br>**Funded studies**<br>Enter a statement with the following details:<br>• Initials of the authors who received each award<br>• Grant numbers awarded to each author<br>• The full name of each funder<br>• URL of each funder website<br>• Did the sponsors or funders play any role in the study design, data collection and analysis, decision to publish, or preparation of the manuscript?<br>• **NO** - Include this sentence at the end of your statement: *The funders had no role in study design, data collection and analysis, decision to publish, or preparation of the manuscript.*<br>• **YES** - Specify the role(s) played.<br><br>* typeset	
**Competing Interests**<br><br>Use the instructions below to enter a competing interest statement for this submission. On behalf of all authors, disclose any competing interests that could be perceived to bias this work—acknowledging all financial support and any other relevant financial or non-financial competing interests.<br><br>This statement is required for submission and **will appear in the published article** if the submission is accepted. Please make sure it is accurate and that any funding sources listed in your Funding Information later in the submission form are also declared in your Financial Disclosure statement.	The authors have declared that no competing interests exist.

View published research articles from *PLOS ONE* for specific examples.<br><br>**NO authors have competing interests**<br><br>Enter: *The authors have declared that no competing interests exist.*<br><br>**Authors with competing interests**<br><br>Enter competing interest details beginning with this statement:<br><br>*I have read the journal's policy and the authors of this manuscript have the following competing interests: [insert competing interests here]*<br><br>* typeset	
**Ethics Statement**<br><br>Enter an ethics statement for this submission. This statement is required if the study involved:<br><br>• Human participants<br>• Human specimens or tissue<br>• Vertebrate animals or cephalopods<br>• Vertebrate embryos or tissues<br>• Field research<br><br>Write "N/A" if the submission does not require an ethics statement.<br><br>General guidance is provided below. Consult the submission guidelines for detailed instructions. **Make sure that all information entered here is included in the Methods section of the manuscript.**	N/A

**Format for specific study types**<br><br>**Human Subject Research (involving human participants and/or tissue)**<br>• Give the name of the institutional review board or ethics committee that approved the study<br>• Include the approval number and/or a statement indicating approval of this research<br>• Indicate the form of consent obtained (written/oral) or the reason that consent was not obtained (e.g. the data were analyzed anonymously)<br><br>**Animal Research (involving vertebrate animals, embryos or tissues)**<br>• Provide the name of the Institutional Animal Care and Use Committee (IACUC) or other relevant ethics board that reviewed the study protocol, and indicate whether they approved this research or granted a formal waiver of ethical approval<br>• Include an approval number if one was obtained<br>• If the study involved *non-human primates*, add *additional details* about animal welfare and steps taken to ameliorate suffering<br>• If anesthesia, euthanasia, or any kind of animal sacrifice is part of the study, include briefly which substances and/or methods were applied<br><br>**Field Research**<br><br>Include the following details if this study involves the collection of plant, animal, or other materials from a natural setting:<br>• Field permit number<br>• Name of the institution or relevant body that granted permission	
**Data Availability**<br><br>Authors are required to make all data underlying the findings described fully available, without restriction, and from the time of publication. PLOS allows rare exceptions to address legal and ethical concerns. See the PLOS Data Policy and FAQ for detailed information.	Yes - all data are fully available without restriction

Powered by Editorial Manager® and ProduXion Manager® from Aries Systems Corporation

A Data Availability Statement describing where the data can be found is required at submission. Your answers to this question constitute the Data Availability Statement and **will be published in the article**, if accepted.<br><br>**Important:** Stating 'data available on request from the author' is not sufficient. If your data are only available upon request, select 'No' for the first question and explain your exceptional situation in the text box.<br><br>Do the authors confirm that all data underlying the findings described in their manuscript are fully available without restriction?	
**Describe where the data may be found in full sentences. If you are copying our sample text, replace any instances of XXX with the appropriate details.**<br><br>• If the data are **held or will be held in a public repository**, include URLs, accession numbers or DOIs. If this information will only be available after acceptance, indicate this by ticking the box below. For example: *All XXX files are available from the XXX database (accession number(s) XXX, XXX.).*<br>• If the data are all contained **within the manuscript and/or Supporting Information files**, enter the following: *All relevant data are within the manuscript and its Supporting Information files.*<br>• If neither of these applies but you are able to provide **details of access elsewhere**, with or without limitations, please do so. For example:<br><br>*Data cannot be shared publicly because of [XXX]. Data are available from the XXX Institutional Data Access / Ethics Committee (contact via XXX) for researchers who meet the criteria for access to confidential data.*<br><br>*The data underlying the results presented in the study are available from (include the name of the third party*	All relevant data are within the manuscript and its Supporting Information files.

Powered by Editorial Manager® and ProduXion Manager® from Aries Systems Corporation

and contact information or URL).<br>• This text is appropriate if the data are owned by a third party and authors do not have permission to share the data.<br><br>* typeset	
Additional data availability information:	

Manuscript

Click here to access/download;Manuscript;Manuscript_R.A.S.Rathnayake.doc

Genetic diversity, phylogenetic and phylogeographic analysis of *Anopheles culicifacies* species complex using ITS2 and COI sequences

R.A.S. Rathnayake[1], Methsala Wedage[2], L.S. Muthukumarana[1], B.G.D.N.K. De Silva[1,2*]

1 Department of Zoology, Faculty of Applied Sciences, University of Sri Jayewardenepura, Nugegoda, Sri Lanka.

2 Genetics and Molecular Biology Unit, Faculty of Applied Sciences, University of Sri Jayewardenepura, Nugegoda, Sri Lanka

***** nissanka@sci.sjp.ac.lk

Abstract

Anopheles culicifacies is the major vector of malaria in Sri Lanka and the Indian subcontinent which is characterized as a species complex with five sibling species provisionally designated as A, B, C, D and E. The current study was carried out to understand the phylogenetic and phylogeographic relationships between the sibling species of the species complex while observing their genetic diversity and genetic differentiation. The available ITS2 and COI sequences of *An. culicifacies* species complex reported from different geographical locations at the NCBI public database were used for the analysis. Bayesian likelihood trees were generated for the phylogenetic analysis. The divergence of the species complex was obtained from the Bayesian phylogeographic model in BEAST. There were two clades of the sibling species of *An. culicifacies* species complex as A, D and B, C and E in both phylogenetic and phylogeographic analysis using ITS2 sequences. Based on the highly divergent COI sequences and the high mutation rate of the mitochondrial genome, there were many clades in both phylogenetic and phylogeographic analysis using COI sequences. The diversification of *An. culicifacies* species complex was obtained as ranging from 20.25 to 24.12 Mya and 22.37 to 26.22 Mya based on ITS2 and COI phylogeographic analysis respectively. There was a recent diversification of the sibling species A and D than the sibling species B, C and E. Low haplotype diversity was observed in the sequences reported from Sri Lanka in both ITS2 and COI analysis that can be due to bottlenecks resulting from the intense malaria control efforts. A high genetic differentiation was achieved for some populations due to the large geographical distance. A high genetic diversity of *An. culicifacies* species complex was suggested through the obtained results implying the possibility of maintaining a relatively high effective population size despite the vector control efforts.

Keywords: *Anopheles culicifacies*, ITS2, COI, Phylogeny, Phylogeography

Introduction

Anopheles culicifacies Giles 1901 is the major vector of malaria in Sri Lanka[1, 2]. It belongs to the order Diptera, sub-order Nematocera, family Culicidae, genus *Anopheles*, subgenus Cellia and series Myzomyia [3]. *An. culicifacies* has a wide distribution that extends from Ethiopia, Iran and Yemen westward to Cambodia, Vietnam, Laos and China eastward whereas the Indian subcontinent towards the northern part and Sri Lanka, and Thailand towards the southern part [4, 5]. In 1977, *An. culicifacies sensu lato* (s.l.) was colonized initially by the National Institute of Malaria Research (previously Malaria Research Centre) in the laboratory [3]. It has been characterized as a species complex with five sibling species provisionally designated as A, B, C, D and E [2], [3] by using cytogenetic methods. The sibling species of the complex are morphologically similar and reproductively isolated. Only sibling species B and E are reported from Sri Lanka while all the members of the species complex are found in India [2], [6].

According to the polytene chromosome diagnostic banding pattern similarities between the sibling species A and D are identified as closely related to each other than the sibling species B, C and E. However, the sibling species B, C and E are known to be closely related to each other [7]. The sequence differences of 28S-D3 rDNA domains and ITS2 rDNA region also supported the classification of the species complex into two groups A, D and B, C, E [4], [illegible], [9]. Vectorial capacity, differences in biting and resting behavior, resistance to insecticides, and susceptibility to malaria parasites of each sibling species vary [10]. Species A, B, C and D are mainly zoophilic and show low anthropophilic index (1-4%) than species E (80%) [11]. However, species A and E are considered as main vectors in the Indian subcontinent than species C and D whereas species B is considered as poor or non-vector [12]. It has been recorded so far that only sibling species B and E are present in Sri Lanka where species E plays the role as major vector [13].

Based on the limited sequence data available at that time it was proposed that the sibling species A and B evolved from a common ancestor. The sibling species D is considered as diverged from sibling species A and the sibling species C from sibling species B in a later period. The origin and distribution of sibling species E persist unclear [9].

The evolutionary potential and history of species are reflected from the genetic diversity and phylogenetic diversity. A higher genetic diversity implies a higher evolutionary potential and a vast ability to respond to environmental changes [14]. A depth understanding of how species evolve through genetic changes can be obtained through phylogenetic analysis and it provides the pathway that connects extant species with their ancestral origin, as well as allows us to predict the genetic divergence that may occur in the future. The phylogeography can be considered as the conceptual bridge connecting processes acting on populations with evolutionary patterns detectable among species or higher taxa [15]. These studies allow us to understand the geographic ordination of genotypes and help to understand both biogeography and landscape genetics across a variety of spatial and temporal scales [16].

The aim of this study was to maximum utilization of the data repository in the public domain, National Center for Biotechnological Information (NCBI) to infer the phylogenetic and phylogeographic relationships of *Anopheles culicifacies* species complex using internal transcribed spacer 2 (ITS2) and cytochrome oxidase I (COI) sequences.

Materials and Methods

Data collection

All sequences of ITS2 and COI of *An. culicifacies* species complex available at NCBI public database (www.ncbi.nlm.nih.gov) were used (S1 and S2 Tables). Four sequences of *Aedes aegypti*

were used as an outgroup of phylogenetic analysis and two sequences of *An. subpictus* were used in the phylogeographic analysis (S3 Table). The sequences were retrieved from NCBI public database (www.ncbi.nlm.nih.gov). There were forty ITS2 sequences displayed and five of them (AJ519494, AJ519493, AJ519492, AJ518810, and AJ534646) were removed from the analysis as it was COII sequences although it was displayed as ITS2. There were seventy seven COI sequences available in the NCBI public database and all the sequences were used for the analysis. The sequences reported countries were indicated in the map of Southeast Asia below (Fig 1).

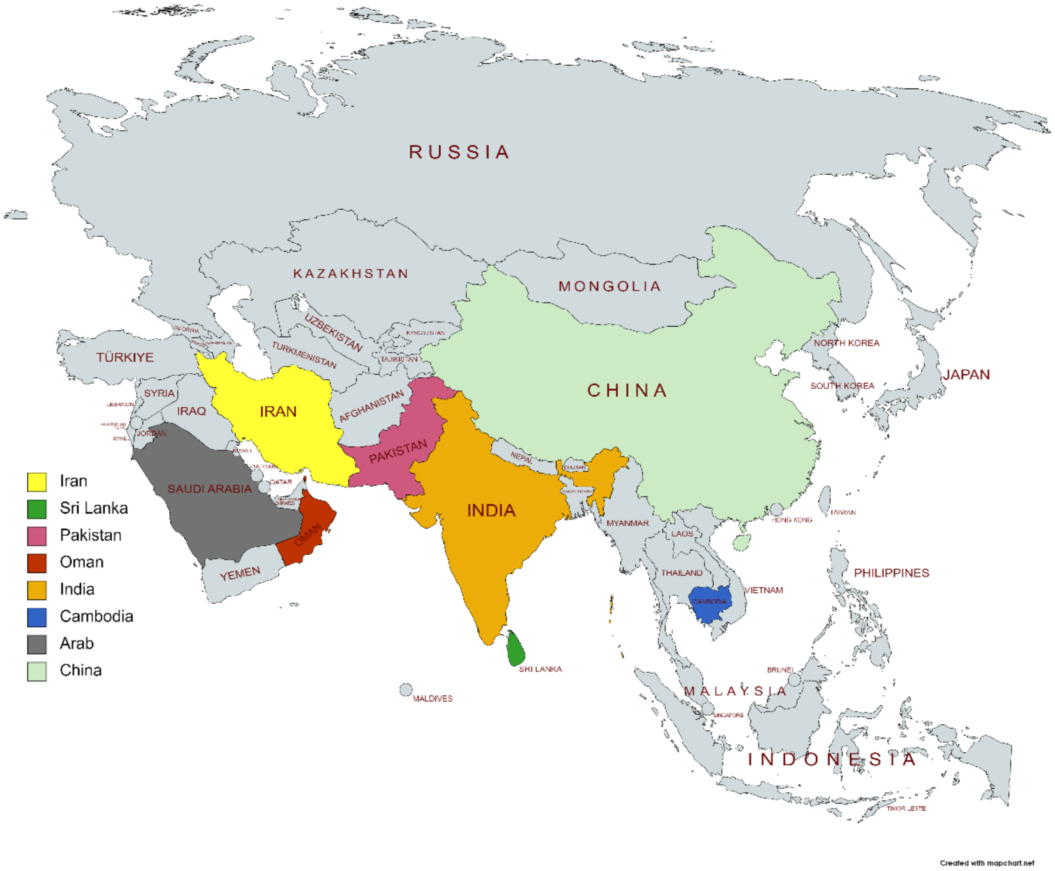

Fig 1. Map of South-East Asia indicating the sequences reported countries

Sequence alignment

All the downloaded sequences of ITS2 and COI were aligned using ClustalW in MEGA5.2.2 software separately [17]. Two separate alignment files were obtained for ITS2 and COI sequences

sets. The sequences of both ITS2 and COI alignment files were trimmed separately to obtain a consistent region as the length of the sequences are different [18]. The final length of ITS2 aligned sequences was 439bp and COI aligned sequences were 450bp.

Phylogenetic analysis

The FASTA format trimmed files of ITS2 & COI after aligning were converted to Nexus alignment (.nex) and Phylip alignment (.phy) file formats by Geneious Prime 2022.1.1 software. The best schemes of models of evolution for ITS2 and COI sequences were obtained by using PartitionFinderV1.1.1_Windows software shown in Table 1. Phylogenetic trees of ITS2 and COI were generated separately using obtained best models of evolution in MrBayes-3.2.5_WIN32_x86: Bayesian inference of Phylogeny software.

Table 1. Best models of evolution obtain for ITS2 and COI sequences

Region/ Gene	Fragment length	Coding positions	Best model of evolution
ITS2	439bp	1 – 439	K80
		2 – 439	JC
		3 – 439	JC
COI	450bp	1 – 450	HKY + G
		2 – 450	HKY + G
		3 – 450	HKY + G

Haplotype Networks construction using ITS2 and COI sequences

DNA Sequences Polymorphism software (DnaSP) (Version 5.10.01) was used to assign coding regions, and genetic code, define sequence sets based on countries and obtain haplotype diversities in each geographical region. Network 10.2 software was used to determine the spatial distribution of haplotypes of ITS2 and COI sequences of *An. culicifacies* using the median-joining method. Arlequin version 3.5.2.2 software was used to obtain the haplotype frequencies of each country [19].

Determination of genetic diversity

Genetic diversity of ITS2 and COI sequences *An. culicifacies* species complex was obtained for the total number of sites (excluding sites with gaps / missing data) [20]. The number of variable sites, GC content, the total number of mutations, number of haplotypes, haplotype diversity, nucleotide diversity and neutrality tests of Tajima's D, Fu and Li's D* test statistic, Fu and Li's F* test statistic, Fu's Fs statistic were obtained for both ITS2 sequences and COI sequences using DnaSP v5 software.

Determination of genetic differentiation

Genetic differentiation of ITS2 and COI sequences *An. culicifacies* species complex among populations of the study was estimated based on fixation index (F_{ST}) values using DnaSP v5 software [21].

Phylogeographic analysis

Phylogeographic analysis using ITS2 and COI sequences following the Bayesian phylogeographic model was carried out in BEAST v1.8.2 software [22, 23]. The diversification date of the most

recent common ancestor (MRCA) of the *Anopheles* genus at 83.23 Mya with a 95% credibility interval ranging from 54.33 to 115.88 Mya [24] was used to calibrate the node ages which was dated using the *Drosophila*-*Anopheles* divergence at 260 Mya suggested by Gaunt and Miles [25].

Results

Phylogenetic analysis of *An. culicifacies* species complex using ITS2 sequences

The generated ITS2 Bayesian likelihood tree was dispersed into two main distinct clades and each clade evolved separately having a different genetic structure for each (Fig 2). The sibling species B, C and E were included in clade 1 and the sibling species A and D were included in clade 2. The sibling species of clade 1 were reported from geographical areas of India, Sri Lanka and Cambodia and China. The sibling species of clade 2 were reported from India and Iran.

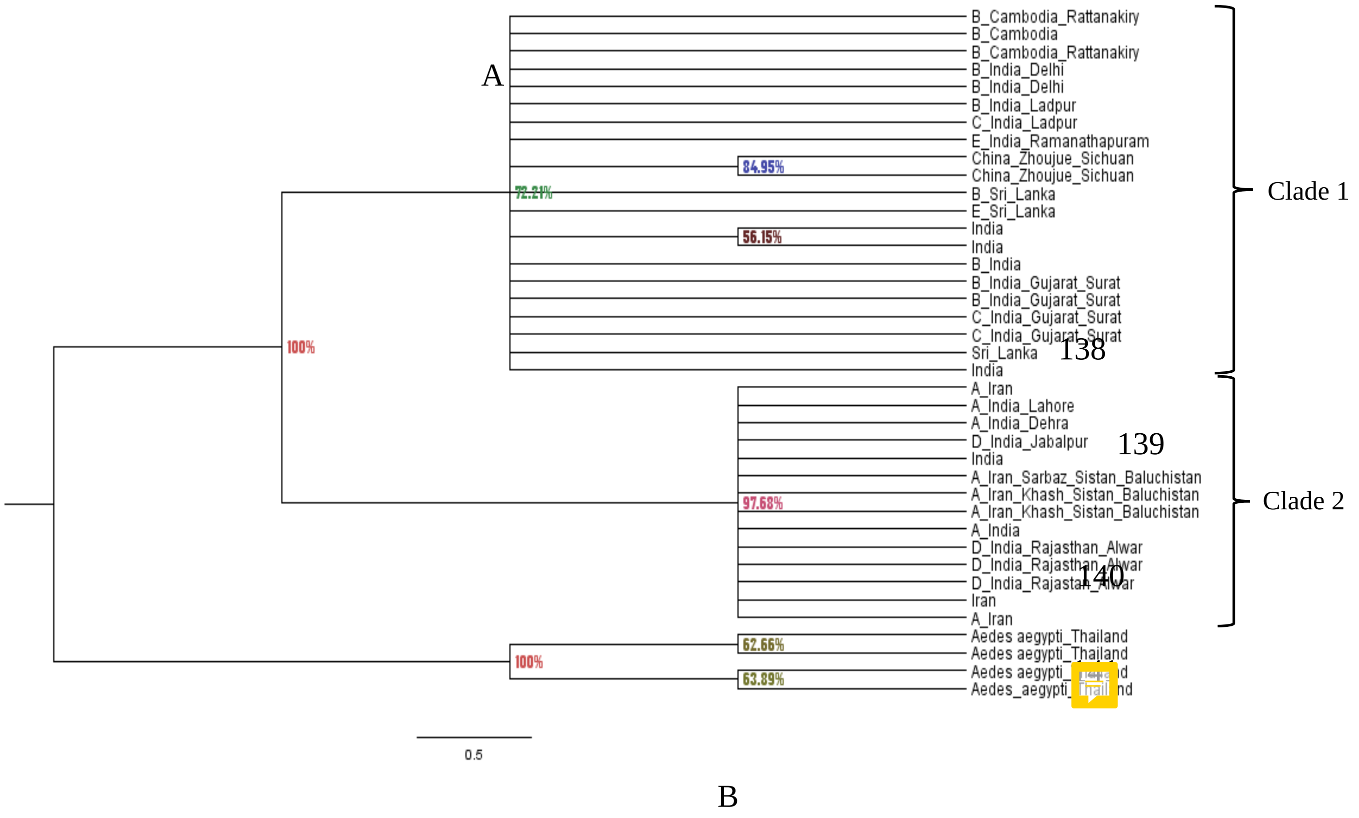

Fig 2. Bayesian likelihood tree generated by MrBayes-3.2.5_WIN32_x86 software using ITS2 sequences of *An. culicifacies*. 35 sequences, 439 characters, 460 000 generations, 2 mcmc runs. Percentages on the node labels are Bayesian posterior probabilities (PP). *Ae. aegypti* was used as the out-group. GenBank accession numbers of ITS2 sequences included in clade 1, 2 and outgroup were listed in S4 Table.

The branch length of clade 2 was greater than the branch length of clade 1 implying that sibling species of A and D of clade 2 might have more divergence than the sibling species of B, C and E in clade 1 from their most recent common ancestor (MRCA). All sibling species of A, B, C, D and E were reported in India. The sibling species B and E were reported in Sri Lanka, only sibling species A was reported from Iran and sibling species B alone was reported from Cambodia.

According to the Fig 1, the posterior probability value in the node of clade 1 was 72.21% (node A). The posterior probability value in the node of clade 2 was 97.68% (node B). As the clade 2 posterior probability value is close to 100 than the clade 1 value, the branching pattern of clade 2 can be considered as statistically more reliable than clade 1.

Phylogenetic analysis of *An. culicifacies* species complex using COI sequences

The tree produced from the phylogenetic analyses of COI sequences (Fig 3) was shown a different topology than the ITS2 Bayesian likelihood tree. The Bayesian likelihood tree of COI sequences was broadly dispersed into two in the first branching point with a posterior probability value of 100% in the branching point (node A). Four clades can be distinguished and each clade was separately evolved having a different genetic structure for each. The clade 1 to 3 were more closely related to each other than clade 4 with a MRCA representing node B with a posterior probability value of 74.73%.

When considering clades 1, 2 and 3, there was a more close relationship seen between clades 2 and 3 having MRCA representing node C with a posterior probability value of 100% implying that the branching pattern was also more statistically reliable. The clade 4 was identified as a derived clade. There was a sister relationship formed by the clade 4 with other clades with its position supported by a high posterior probability value of 86.05%.

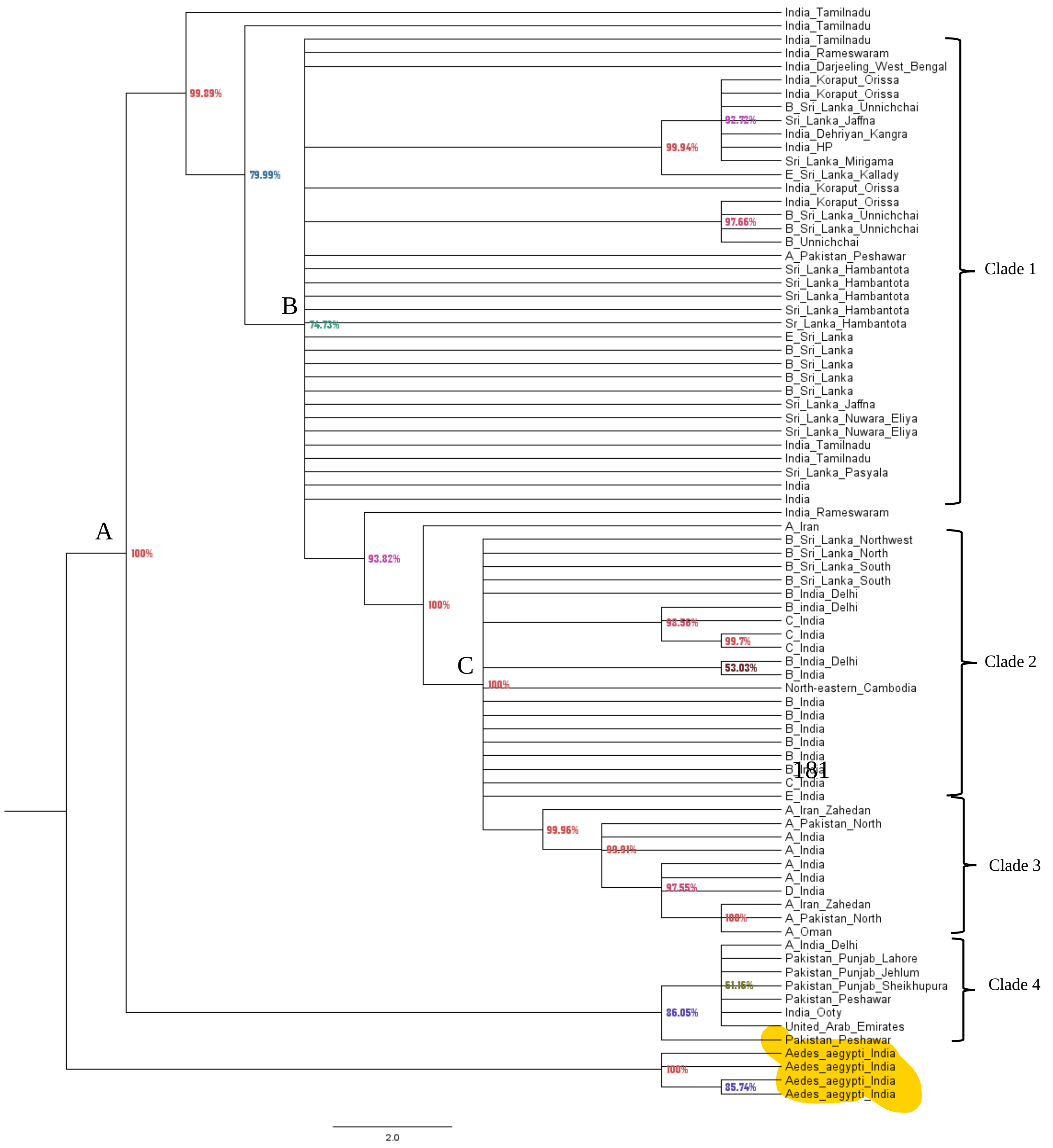

India_Tamilnadu
India_Tamilnadu
India_Tamilnadu
India_Rameswaram
India_Darjeeling_West_Bengal
India_Koraput_Orissa
India_Koraput_Orissa
B_Sri_Lanka_Unnichchai
Sri_Lanka_Jaffna
India_Dehriyan_Kangra
India_HP
Sri_Lanka_Mirigama
E_Sri_Lanka_Kallady
India_Koraput_Orissa
India_Koraput_Orissa
B_Sri_Lanka_Unnichchai
B_Sri_Lanka_Unnichchai
B_Unnichchai
A_Pakistan_Peshawar
Sri_Lanka_Hambantota
Sri_Lanka_Hambantota
Sri_Lanka_Hambantota
Sri_Lanka_Hambantota
Sr_Lanka_Hambantota
E_Sri_Lanka
B_Sri_Lanka
B_Sri_Lanka
B_Sri_Lanka
B_Sri_Lanka
Sri_Lanka_Jaffna
Sri_Lanka_Nuwara_Eliya
Sri_Lanka_Nuwara_Eliya
India_Tamilnadu
India_Tamilnadu
Sri_Lanka_Pasyala
India
India
India_Rameswaram
A_Iran
B_Sri_Lanka_Northwest
B_Sri_Lanka_North
B_Sri_Lanka_South
B_Sri_Lanka_South
B_India_Delhi
B_india_Delhi
C_India
C_India
C_India
B_India_Delhi
B_India
North-eastern_Cambodia
B_India
B_India
B_India
B_India
B_India
B_India
C_India
E_India
A_Iran_Zahedan
A_Pakistan_North
A_India
A_India
A_India
A_India
D_India
A_Iran_Zahedan
A_Pakistan_North
A_Oman
A_India_Delhi
Pakistan_Punjab_Lahore
Pakistan_Punjab_Jehlum
Pakistan_Punjab_Sheikhupura
Pakistan_Peshawar
India_Ooty
United_Arab_Emirates
Pakistan_Peshawar
Aedes_aegypti_India
Aedes_aegypti_India
Aedes_aegypti_India
Aedes_aegypti_India
99.89%
79.99%
92.72%
99.94%
97.66%
74.73%
100%
93.82%
100%
98.56%
99.7%
53.03%
100%
99.96%
99.91%
97.55%
100%
61.16%
86.05%
100%
85.74%
A
B
C
Clade 1
Clade 2
Clade 3
Clade 4
2.0

Fig 3. Bayesian likelihood tree generated by MrBayes-3.2.5_WIN32_x86 software using COI sequences of *An. culicifacies*. 77 sequences, 450 characters, 1 640 000 generations, 2 mcmc runs. Percentages on the node labels are Bayesian posterior probabilities (PP). *Ae. aegypti* was used as the out-group. GenBank accession numbers of COI sequences included in Clade 1 to 4 and outgroup were listed in S5 Table.

The sibling species B, C and E were included in clade 2 from India (sibling species B, C, E), Sri Lanka (sibling species B) and North-eastern Cambodia. The sibling species A and D were included in clade 3 where A from Iran, India, Pakistan and Oman and D from India. The sibling species from India, Pakistan and the United Arab Emirates were included in clade 4.

According to the Fig 3, the branch length of clade 3 was greater than the branch length of clade 2 implying the sibling species of A and D of clade 3 might have more genetic change (or divergence) than the sibling species of B, C and E in clade 2 from their MRCA. That result was the same with ITS2 phylogenetic analysis which was that the sibling species A and D might have more genetic divergence than sibling species B, C and E from their MRCA.

ITS2 haplotype network of *An. culicifacies* species complex

The ITS2 haplotypes of *An. culicifacies* species complex reported countries were shown in Fig 4 and ITS2 haplotype network was shown in Fig 5. There was a total of 20 haplotypes recorded from Iran, Cambodia, India, China and Sri Lanka based on the 35 ITS2 sequences used in the analysis. The haplotype frequencies and haplotype diversities (Hd) in each population were listed in S6 Table. There were 3 shared haplotypes named as H_2, H_11 and H_14 and the other remaining haplotypes were private haplotypes.

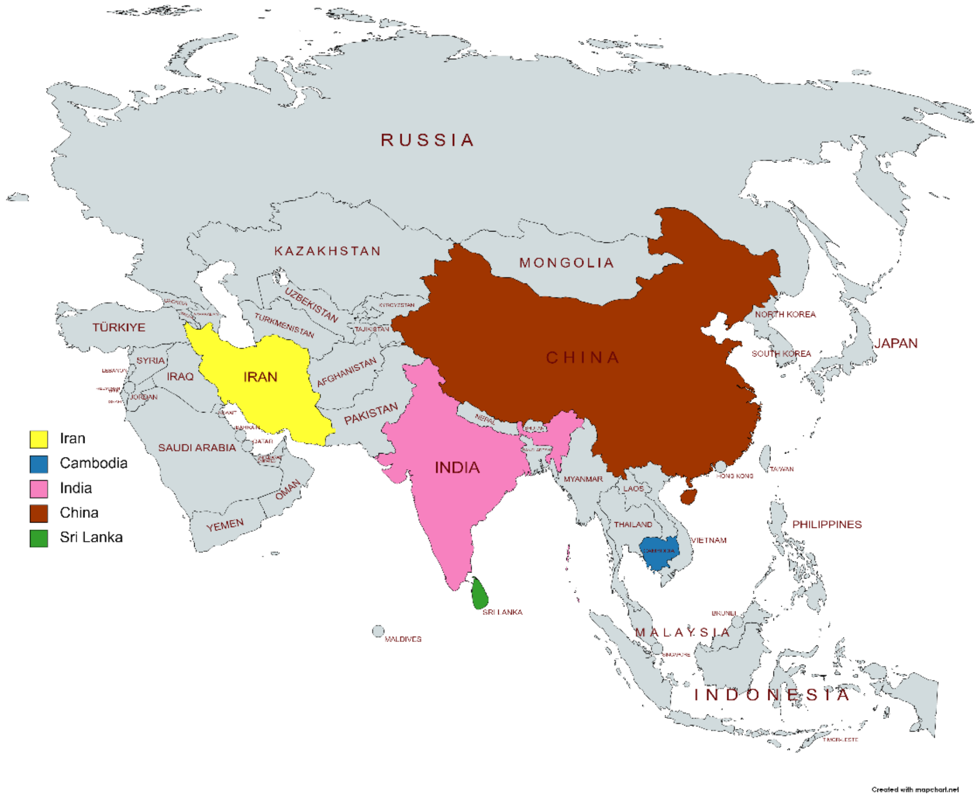

Fig 4. Map of South-East Asia indicating the haplotypes of ITS2 reported countries

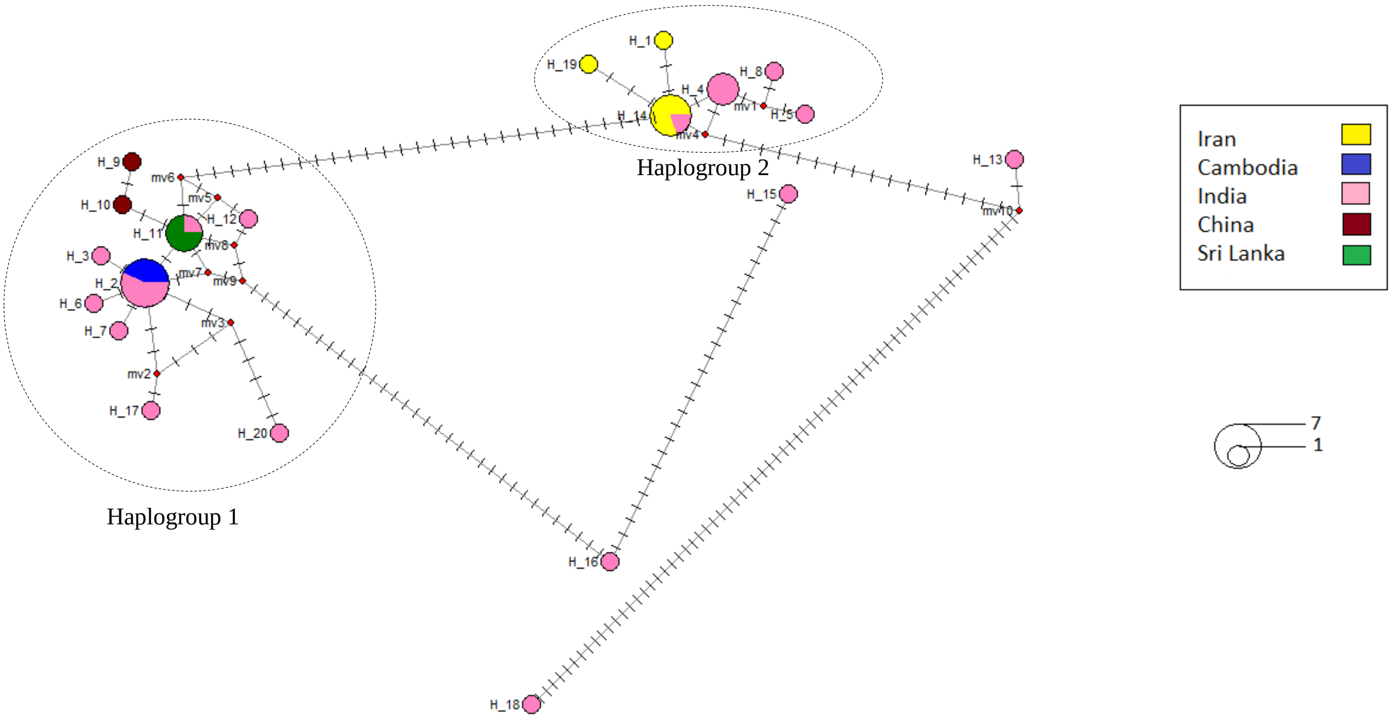

Fig 5. ITS2 haplotype network of *An. culicifacies* species complex (20 haplotypes) generated using DnaSP v5 software and Network 10.2 software. The size of a circle indicates the relative frequency of sequences belonging to a certain sequence type. Each color indicates a different geographic area. The distance between two haplotypes includes no. of mutations between them. Red colored dots indicate median vectors.

The highest number of private haplotypes (13) were recorded from India (Hd= 0.729) while there were no private haplotypes from Cambodia and Sri Lanka each having one shared haplotype with India. Hd values were zero in Cambodia, China and Sri Lanka. Any of the haplotypes from China was not shared between other localities.

There were two main haplogroups. The haplogroup 1 was composed of haplotypes from India, Cambodia, Sri Lanka and China. There were predominantly private haplotypes from India with its base made of two shared haplotypes H_2 and H_11. The regional ancestor of the haplogroup 1 was H_2 which is a shared haplotype between India and Cambodia that was understood as the genetic backup of the haplogroup 1.

The haplogroup 2 was made up of private haplotypes from Iran and India with one shared haplotype H_14 that was shared between those two populations. The regional ancestor of the haplogroup 2 was H_14 which is a shared haplotype between Iran and India.

The highest distance was shown between the H_18 and H_13 haplotypes of India having highest no. of mutations between them.

COI haplotype network of *An. culicifacies* species complex

The COI haplotypes of *An. culicifacies* species complex reported countries were shown in Fig 6 and the COI haplotype network was shown in Fig 7. There was a total of 55 haplotypes recorded

from Iran, Sri Lanka, Pakistan, Oman, India, Cambodia and United Arab based on the United Arab Emirates 77 COI sequences used in the analysis.

The haplotype frequencies and haplotype diversities (Hd) in each population were listed in Table S7. There were 4 shared haplotypes named as Hap_4, Hap_13, Hap_16 and Hap_32 and the remaining ones were private haplotypes.

The highest number of private haplotypes (26) were recorded from India (Hd= 0.980) while the least number of private haplotypes (one from each) were recorded from Oman, United Arab Emirates and Cambodia. The lowest Hd value was obtained from Sri Lanka (Hd=0.957). The haplotypes of United Arab Emirates, Iran, Cambodia and Oman were not shared between other localities.

There were four main haplogroups. The haplogroup 1 was made up of haplotypes from India, Sri Lanka and Pakistan. There were predominantly private haplotypes from Sri Lanka and two shared haplotypes (Hap_16, Hap_32) that were shared between India and Sri Lanka. The regional ancestor of the haplogroup 1 was identified as Hap_40 from India.

The haplogroup 2 was made up of haplotypes from India, Sri Lanka and Cambodia. There were predominantly private haplotypes from India and no shared haplotypes. The regional ancestor of the haplogroup 2 was identified as Hap_9 of India.

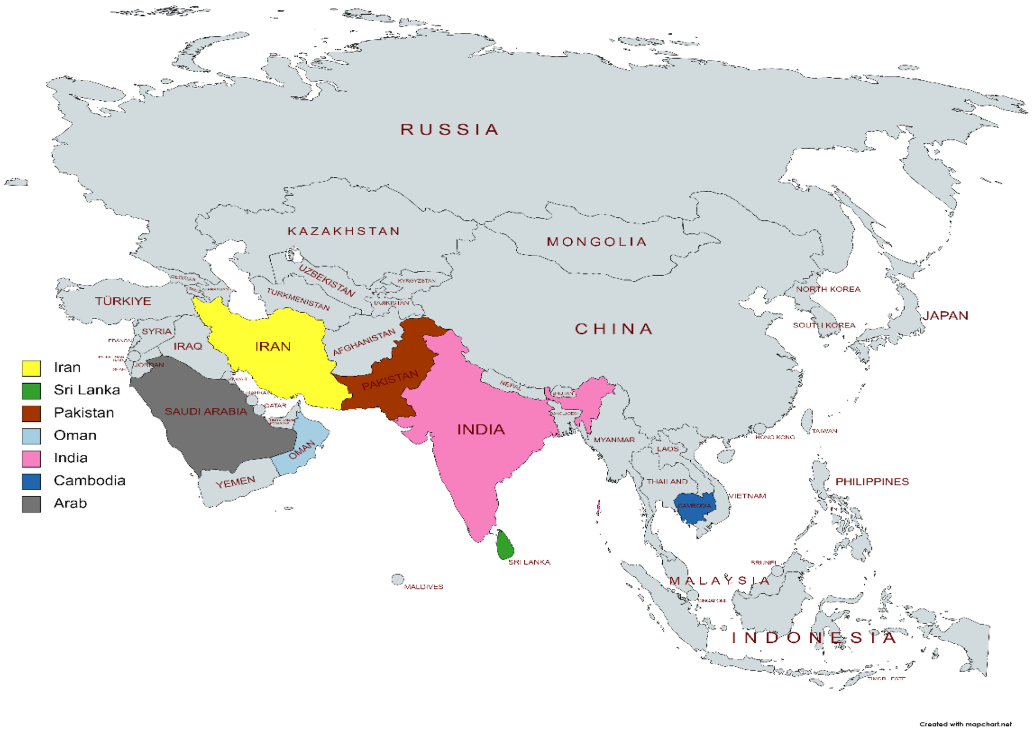

Fig 6. Map of South-East Asia indicating the haplotypes of COI reported countries

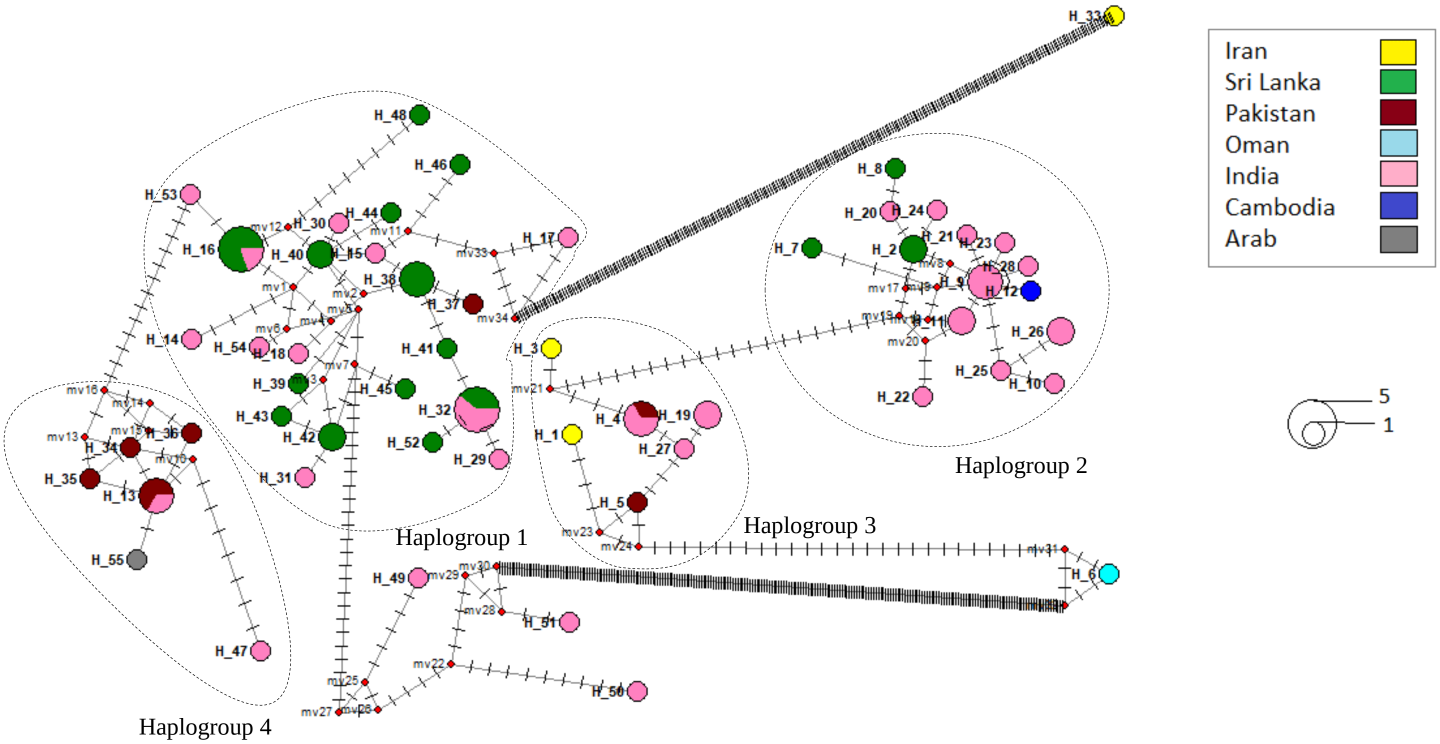

Fig 7. COI sequences reported countries and COI haplotype network of *An. culicifacies* species complex (55 haplotypes) generated using DnaSP v5 software and Network 10.2 software. The size of a circle indicates the relative frequency of sequences belonging to a certain sequence type. Each color indicates a different geographic area. The distance between two haplotypes includes no. of mutations between them. Red colored dots indicate median vectors.

The haplogroup 3 was made up of haplotypes from India, Pakistan and Iran. There was one shared haplotype Hap_4 that was shared between India and Pakistan. The regional ancestor of the haplogroup 3 was identified as Hap_4 which is a shared haplotype between Pakistan and India.

The haplogroup 4 was composed of haplotypes from India, Pakistan and the United Arab Emirates. There were predominantly private haplotypes from Pakistan and one shared haplotype Hap_13 that was shared between India and Pakistan. The regional ancestor of the haplogroup 4 was identified as Hap_13 which is a shared haplotype between Pakistan and India.

Determination of genetic diversity

The genetic diversity of *An. culicifacies* species complex based on ITS2 and COI sequences was shown in Table 2 obtained using DnaSP Version 5.10.01 software.

Table 2. Number of variable sites (S), haplotypes (h), nucleotide diversity (π), haplotype diversity (Hd), the average number of nucleotide differences (k), Tajima's D, Fu and Li's D, Fu and Li's F, Fu's Fs statistic based on COI and ITS2 sequences of *An. culicifacies* using DnaSP v5 software.

Region/Gene	Sample Size	S	h	π	Hd	k	Tajima's D	Fu and Li's D	Fu and Li's F	Fu's Fs statistic
ITS2	35	28	20	0.0252	0.661	8.5916	0.92220**	-1.24558**	-0.61994**	5.755
COI	77	301	55	0.2752	0.981	104.0123	0.82540**	-2.48524*	-1.32233**	5.036

* Significant $P<0.05$, **Not significant $P>0.10$

The genetic diversity of *An. culicifacies* species complex that was measured at both ITS2 and COI sequences using DnaSP Version 5.10.01 software was shown in Table 2. When considering all sequences of ITS2 and COI, the nucleotide diversity (π), haplotype diversity (Hd), and an average number of nucleotide differences (k) were higher in COI sequences than in ITS2 sequences.

The high haplotype diversity (Hd) values of 0.661 to 0.981 was observed in ITS2 and COI sequences respectively. The nucleotide diversity (π) and the average number of nucleotide differences (k) were higher in the analysis of COI sequences than in ITS2 sequences.

The G+C content was 0.591 at coding positions of 341.00 sites in ITS2 sequences and the total G+C content was 0.305 at coding positions of 378.00 sites in COI sequences. The total number of mutations was higher in COI sequences than in ITS2 sequences having 413 of a total number of mutations in COI and 28 total number of mutations in ITS2.

In both ITS2 and COI analysis statistically non-significant ($P>0.10$) positive values of Tajima's D values were obtained. There were negative values of Fu and Li's F and D and positive values of Fu's Fs statistic in both ITS2 and COI sequence analyses.

Determination of genetic differentiation

Genetic differentiation based on ITS2 sequences of *An. culicifacies* species complex

Pairwise fixation index (F_{ST}) values among populations were obtained using DnaSP v5 software for ITS2 sequences of *An. culicifacies* species complex was shown in Table 3.

Table 0. Pairwise fixation index (F_{ST}) values among populations based on ITS2 sequences of *An. culicifacies* species complex using DnaSP v5 software.

	Iran	Cambodia	India	China	Sri Lanka
Iran	0				
Cambodia	0.98969	0			
India	0.56090	0.32230	0		
China	0.98969	0.00000	0.32230	0	
Sri Lanka	0.98969	0.00000	0.32230	0.00000	0

The common used values for F_{ST} are as follows: 0 to 0.05, small; 0.05 to 0.15, moderate; 0.15 to 0.25, great; values above 0.25 indicate huge genetic differentiation [21].

According to the Table 3, there were F_{ST} values ranging from 0.00000 to 0.98969. The highest F_{ST} values near to 1 (0.98969) were obtained for Iran-Cambodia, Iran-China and Iran-Sri Lanka indicating huge genetic differentiation between populations. Zero F_{ST} values were obtained for Cambodia-China, Cambodia-Sri Lanka and China-Sri Lanka indicating the absence of genetic subdivision between the considered populations.

Genetic differentiation based on COI sequences of *An. culicifacies* species complex

Pairwise fixation index (F_{ST}) values among populations were obtained using DnaSP v5 software for COI sequences of *An. culicifacies* species complex was shown in Table 4.

Table 4. Pairwise fixation index (F_{ST}) values among populations based on COI sequences of *An. culicifacies* species complex using DnaSP v5 software.

	Iran	India	Sri Lanka	Pakistan
Iran	0			
India	0.07118	0		
Sri Lanka	0.40577	0.21970	0	
Pakistan	0.28518	0.10061	-0.00263	0

The common used values for F_{ST} are as follows: 0 to 0.05, small; 0.05 to 0.15, moderate; 0.15 to 0.25, great; values above 0.25 indicate huge genetic differentiation [21].

According to the Table 4, there were F_{ST} values ranging from -0.00263 to 0.40577. The highest F_{ST} value (0.40577) was obtained for Iran-Sri Lanka indicating huge genetic differentiation between populations. A negative F_{ST} value was obtained for Sri Lanka-Pakistan. Negative F_{ST} values should be effectively seen as zero values indicating the absence of genetic subdivision between the considered populations when the allele frequencies of two subpopulations are very similar. F_{ST} values above 0.25 indicating huge genetic differentiation were shown in Iran-Sri Lanka, and Iran-Pakistan.

Phylogeographic analysis of *An. culicifacies* species complex using ITS2 sequences

The phylogeographic tree of *An. culicifacies* species complex using ITS2 sequences was shown in Fig 5. According to the Fig 5, the diversification of the *Anopheles* genus was dated at 83.06 Mya (node A) with a 95% HPD ranging from 81.15 to 85.09 Mya in the Cretaceous. The diversification of *An. culicifacies* species complex was dated at 22.19 Mya (node B) with a 95% HPD ranging from 20.25 to 24.12 Mya in the late Miocene.

The species complex was split into two clades as in the phylogenetic analysis of ITS2 sequences. The diversification of clade 1 including sibling species A and D was dated at 16.25 Mya (node C) in Miocene. The sibling species A from Iran and both sibling species A and D from India were included in clade 1. There are two subclades inside clade 1 at node E and F that were dated at 12.11 Mya and 10.85 Mya separately. The diversification of clade 2 including sibling species B, C and E was 19.17 Mya (node D) in Miocene. The sibling species B from Sri Lanka, Cambodia and India, the sibling species C from India and the sibling species E from Sri Lanka were included in clade 2. There are three subclades inside clade 2 at nodes G, H and I that were dated at 8.73 Mya, 16.47 Mya and 16.18 Mya separately.

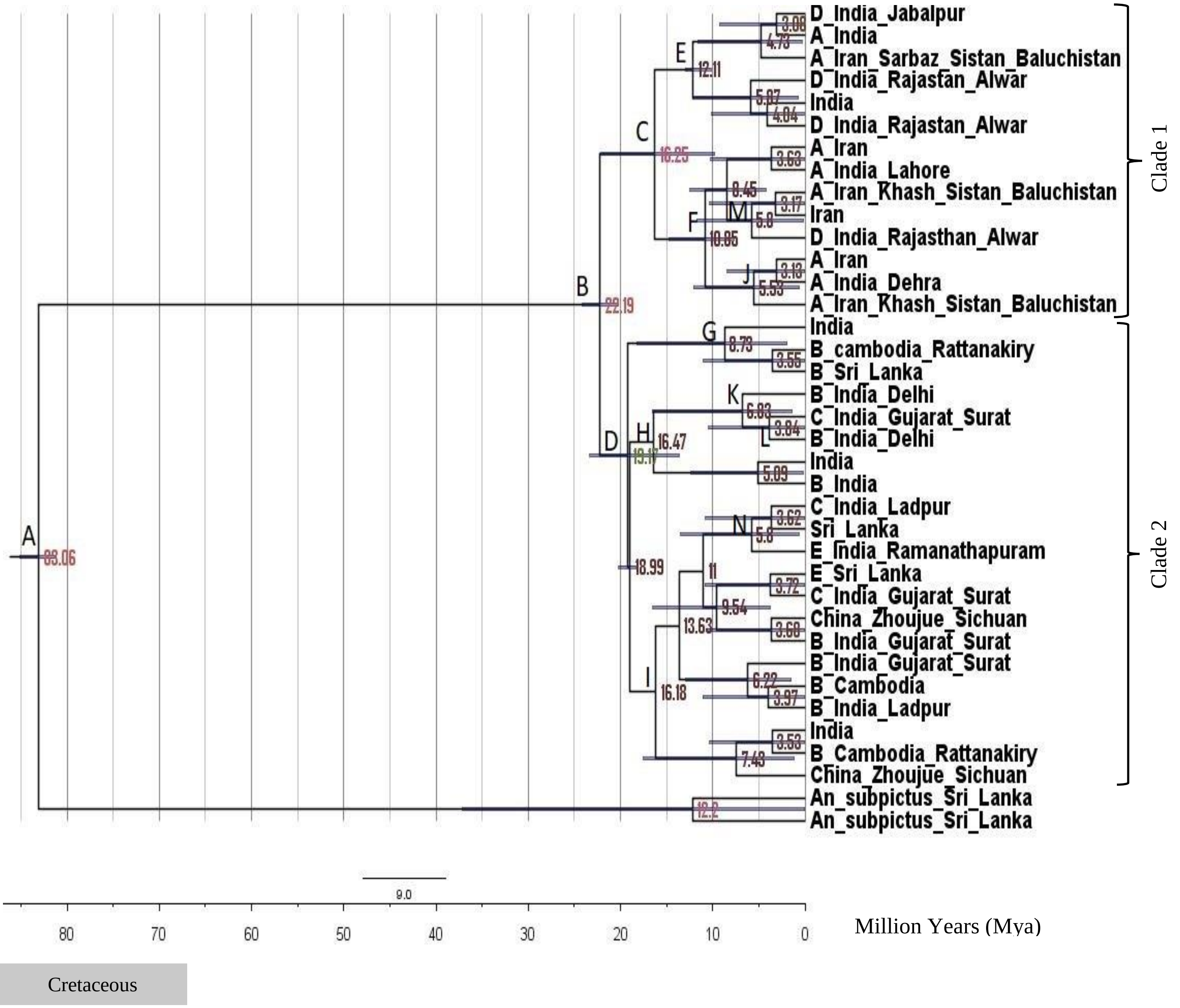

Node	PP	Mean (Mya)	95% HPD (Mya)
A	1	83.06	81.15-85.09
B	1	22.19	20.25-24.12
C	0.963	16.25	9.77-22.12
D	0.217	19.17	13.62-23.34
E	0	12.11	10.17-12.98
F	0	10.85	7.70-14.71
G	0.003	8.73	1.94-18.23
H	0	16.47	19.17-13.62

I	0		
J	0.005	5.53	0.60-12.07
K	0.003	6.83	1.38-16.57
L	0.034	3.84	0.06-10.44
M	0.007	5.80	0.12-11.73
N	0.003	5.80	0.64-13.48

Pala Eoce Olig Mio

Geological Time Scale

Fig 8. Evolutionary timescale for *An. culicifacies* species complex generated by BEAST v1.8.2 software using ITS2 sequences. 35 sequences, 435 characters. *An. subpictus* was used as the out-group. Numbers near the nodes designate the average divergence time estimated (Million Years, Mya). Geological time scale includes Cretaceous, Pala(eocene), Eoce(ne), Olig(ocene) and Mioc(ene) respectively. GenBank accession numbers of ITS2 sequences included in clades 1, 2 and outgroup were listed in S8 Table.

The diversification of the clade 1 including sibling species A and D was more recent than the clade 2 with sibling species B, C and E based on ITS2 phylogeographic analysis. The divergence of ITS2 sequences into clade 1 and clade 2 occurred prior to splitting into sibling species.

The diversification of the clade 1 including sibling species A and D was more recent than the clade 2 with sibling species B, C and E based on ITS2 phylogeographic analysis. The divergence of ITS2 sequences into clade 1 and clade 2 occurred prior to splitting into sibling species.

The diversification times of the earliest members of the sibling species A, B, C, D and E based on the ITS2 phylogeographic tree was listed in Table 5. According to that, the sibling species B can be considered as the earliest evolved sibling species and the sibling species C was the recently evolved sibling species based on ITS2 sequences.

Table 5. Diversification times of earliest members of the sibling species A, B, C, D and E of *An. culicifacies* species complex with countries based on the ITS2 phylogeographic tree generated by BEAST v1.8.2 software.

Sibling species	Diversification (Mya)	Country
A	5.53 (node J)	Iran
B	6.83 (node K)	India
C	3.84 (node L)	India
D	5.80 (node M)	India
E	5.80 (node N)	India

Phylogeographic tree of *An. culicifacies* species complex using COI sequences

The phylogeographic tree of *An. culicifacies* species complex using COI sequences was shown in Fig 6. The split time of the most recent common ancestor of the *Anopheles* genus was obtained as 82.75 Mya in the Cretaceous (node A) with a 95% HPD (Height posterior density) ranging from

80.93 to 85.07 Mya. The split date of *An. culicifacies* species complex was 24.33 Mya shown in node B with a 95% 95% HPD ranging from 22.37 to 26.22 Mya.

There were three clades diversified in the Miocene period. The diversification of clade 1 including sibling species from Iran, India, Pakistan, Oman and Sri Lanka was dated as 17.30 Mya (node C). The diversification of clade 2 and clade 3 was dated as 18.07 Mya (node D) and 17.92 Mya (node E) respectively. The sibling species from Sri Lanka, India and Pakistan were included in clade 2. The sibling species from Iran, India, Sri Lanka, Pakistan and the United Arab Emirates were included in clade 3.

There were three subclades identified within clade 1 arising at nodes F, G and H. The first subclade arising at node F was dated 10.11 Mya including sibling species A and D from India, and sibling species A from Pakistan, Iran and Oman. The second sub clade arising at node G was dated as 9.59 Mya including sibling species B and C from India, and sibling species B from Sri Lanka. The third sub clade arising at node H was dated 11.77 Mya including species B, C and E from India and one specimen from Northeastern Cambodia. There were two sub clades identified within clade 2 arising at node I and J dated at 12.18 Mya and 16.35 Mya separately.

The diversification of sibling species of the species complex mainly occurred in Miocene. The diversification times of the earliest members of the sibling species A, B, C, D and E based on the COI phylogeographic tree were listed in Table 6. According to that, the sibling species A can be identified as the earliest evolved sibling species and the sibling species D was the recently evolved sibling species based on COI sequences.

Node	PP	Mean (Mya)	95% HPD (Mya)
A	1	82.75	80.93-85.07
B	0.98	24.33	22.37-26.22
C	1	17.30	10.36-24.68
D	0.04	18.07	13.24-23.46
E	0.02	17.92	13.17-24.28
F	0.09	10.11	4.02-17.12
G	0.33	9.59	3.19-16.05
H	0.50	11.77	5.79-17.9
I	0.40	12.18	5.45-19.96
J	0.01	16.35	10.37-21.15
K	0.31	7.22	1.40-14.14
L	0.07	6.18	0.74-12.70
M	0.51	3.28	0.11-7.36
N	0.97	8.39	2.83-14.72

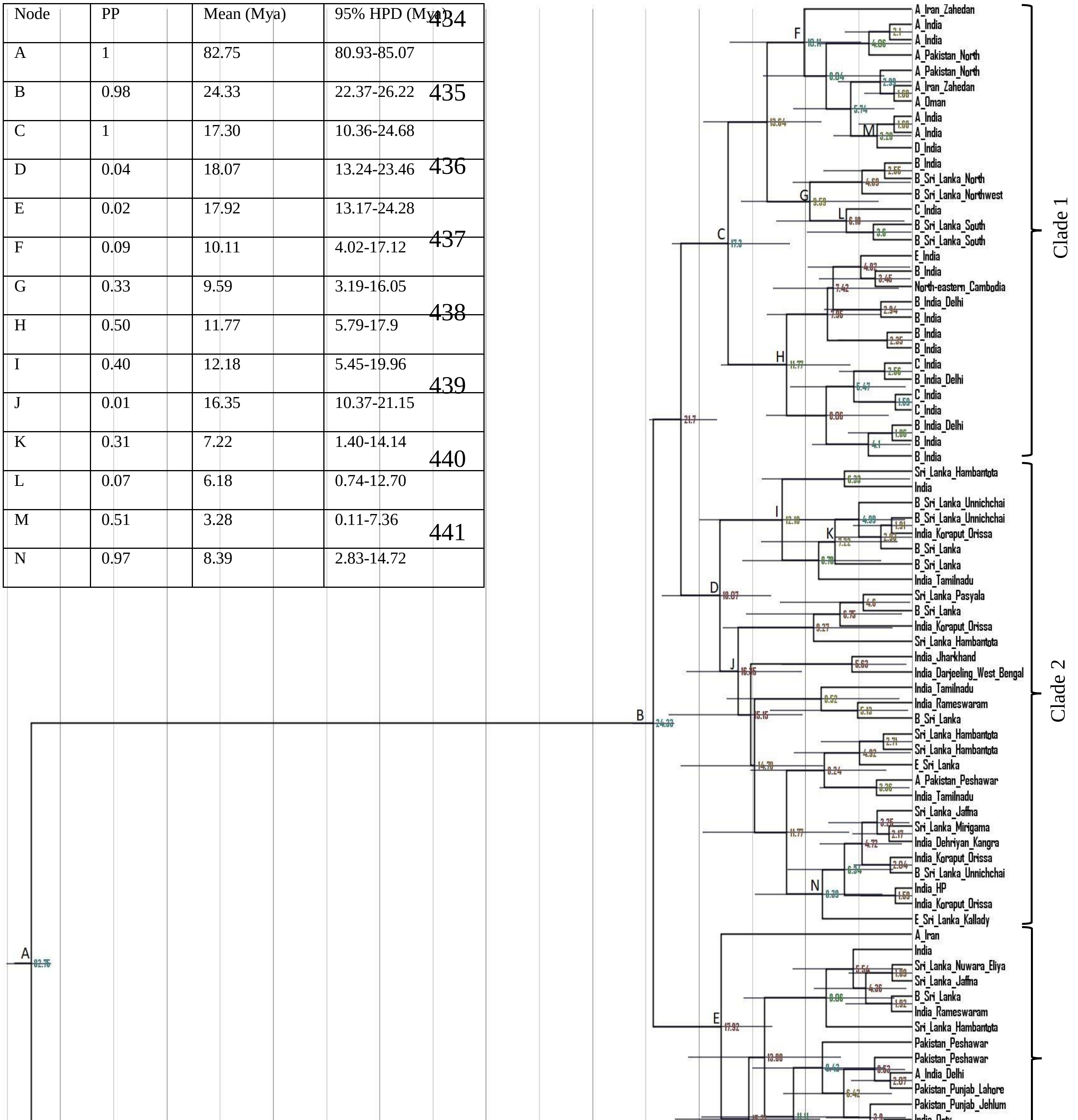

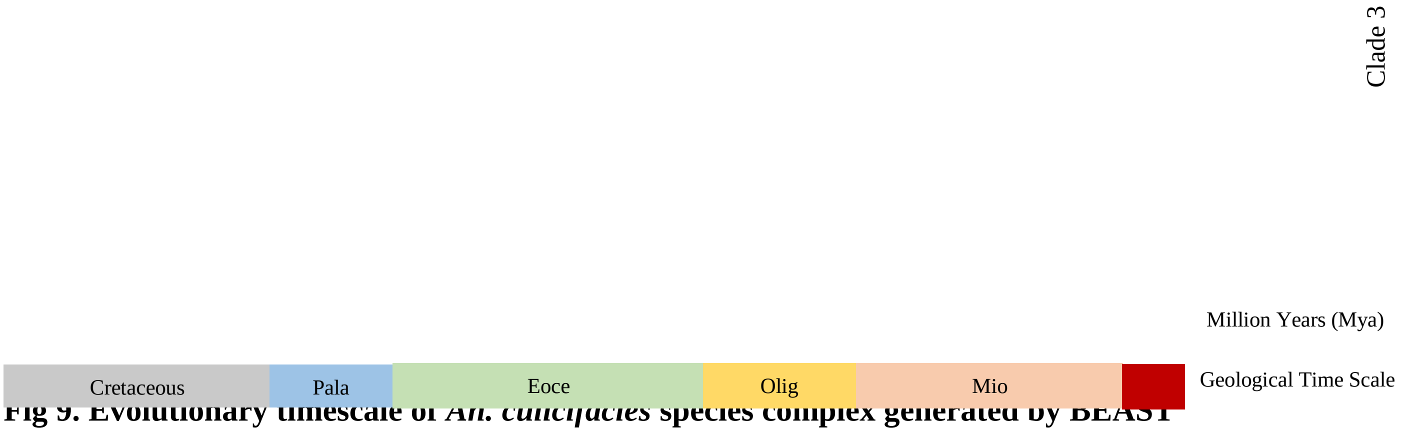

Fig 9. Evolutionary timescale of *An. culicifacies* species complex generated by BEAST v1.8.2 software using COI sequences. Sequences were retrieved from NCBI GenBank, 77 sequences, 450 characters. *An. subpictus* was used as the out-group. Numbers near the nodes designate the average divergence time estimated (Million Years, Mya). Geological time scale includes Cretaceous, Pala(eocene), Eoce(ne), Olig(ocene) and Mioc(ene) respectively. GenBank accession numbers of COI sequences included in clades 1 to 3 and outgroup were listed in S9 Table.

Table 6. Diversification times of earliest members of the sibling species A, B, C, D and E of *An. culicifacies* species complex with countries based on the COI phylogeographic tree generated by BEAST v1.8.2 software.

Sibling species	Diversification (Mya)	Country
A	17.92 (node E)	Iran
B	7.22 (node K)	Sri Lanka
C	6.18 (node L)	India
D	3.28 (node M)	India
E	8.39 (node N)	Sri Lanka

Discussion

Molecular taxonomy allows us to distinguish the mosquito vectors to species or sibling species level. Molecular systematics provides information on genetic diversity and supports to predict their evolution and phylogenetic relationships [26]. The analysis of phylogenetic and phylogeographic relationships, identification of haplotypes in different geographical areas, understanding of genetic diversity and genetic differentiation based on ITS2 and COI sequences of *An. culicifacies* species complex was carried out in this study. The Bayesian likelihood trees generated using ITS2 and COI sequences further supported the sibling species identification and displayed the phylogenetic relationships between sibling species of *An. culicifacies* species complex with well-supported clades with high posterior probability values. In the phylogenetic analysis using ITS2 sequences, the generated Bayesian likelihood tree was dispersed into two main distinct clades including the sibling species B, C, and E in clade 1 and the sibling species A and D in clade 2. A considerable sequence divergence between these two clades and a strict sequence conservation within the members of a given clade was implied.

That result was the same as the results based on the primary and secondary structure of the ITS2 sequences. There are two clades of sibling species as A, D and B, C, and E of the species complex based on the primary and secondary structures of the ITS2 sequences [2].

According to the previous studies, five sibling species differed in polytene chromosome banding sequences or metaphase karyotypes correlated with reproductive barriers that differentiate sibling species A, B, C, D and E [1]. The crossing experiments between sibling species have shown that species A and B, species A and C, species B and D, species C and D produce fertile hybrid females

and sterile hybrid males. There are both pre-mating and post-mating barriers found between species A, species B and species A and species C. There is only a pre mating barrier identified between species B and species C. A bidirectional hybrid male sterility can be seen between species A and B and species B and C [12].

There was a more genetic change (or divergence) observed in the sibling species of A, and D than in the sibling species of B, C, and E after splitting from a common ancestor based on the branch length of the two clades of the ITS2 Bayesian likelihood tree. Another phylogenetic analysis of *An. culicifacies* species complex based on the ITS2 sequences performed using multiple aligned sequences with a neighbour-joining distance method, the species of A and D are under a less selective pressure and evolving faster compared to the sibling species in B, C and E. The members in a certain clade having a near sequence identity without any possibility of cross-breeding suggests that ITS2 regions are undergoing parallel evolution to achieve clade-specific RNA biogenesis [8].

In the phylogenetic analysis using COI sequences, the generated Bayesian likelihood tree was dispersed into four clades producing a tree with a different topology than the ITS2 Bayesian likelihood tree. Only in the clades 2 and 3 of the COI Bayesian likelihood tree which includes the sibling species B, C, and E in the clade 2 and sibling species A, and D in the clade 3 were shown near relatedness to the clades of the ITS2 Bayesian likelihood tree. When considering the branch lengths of the clades 2 and 3, the obtained results were the same as the ITS2 phylogenetic analysis which was the sibling species A and D might have more genetic divergence than the sibling species B, C and E from their MRCA.

The generated COI Bayesian likelihood tree was divided into many clades as highly divergent COI sequences and the mitochondrial genome has a higher mutation rate than the nuclear genome.

According to the generated two haplotype networks, the total number of haplotypes was higher in the COI haplotype network than in the ITS2 haplotype network. It can be due to the haploid mitochondrial genome having a higher mutation rate than the nuclear genome and the occurring variations can produce different haplotypes. The considered number of COI sequences was also higher than the number of ITS2 sequences in this study.

The highest number of private haplotypes was recorded from India with high Hd values in both ITS2 and COI haplotype networks. A large effective population size in this area is represented by this private haplotype richness and the high haplotype diversity. The private haplotypes in a particular geographical area are indicators of a restricted gene flow from that area and a population that is adapted to local conditions [27].

There were lower Hd values obtained from Sri Lankan sequences among the sequences in both ITS2 and COI analysis. The low haplotype diversity that occurred from bottlenecks might have resulted from the intense malaria control efforts, habitat fragmentation and disappearance of vertebrate blood meal sources [27]. But, it can become high through the imported cases resulting in a risk of disease re-emergence in Sri Lanka.

When considering the genetic diversity of *An. culicifacies* species complex based on the ITS2 and COI sequences recorded from all geographical regions, high Hd values were observed in both analyses, suggesting an extensive genetic diversity of the mosquitoes could be due to several facts including a large effective population size of mosquitoes, high gene flow among populations and favorable environmental conditions for mosquito breeding, regardless of control campaigns [28]. This high level of genetic diversity observed in this study implies that the possibility of *An. culicifacies* species complex maintaining a relatively high effective population size despite enormous population fluctuations.

A long-established mosquito population that has not faced recent bottlenecks and the possibility of the presence of genetically distant haplotypes in sympatry was indicated by the higher nucleotide diversity (π) and an average number of nucleotide differences (k) [28] in the analysis of COI sequences than the ITS2 sequences.

The null hypothesis of neutral evolution indicating that the mosquito populations in the study at mutation-drift equilibrium maintained a stable large population was accepted from non-significant Tajima's D values [28]. That was indicated by the non-significant Tajima's D values of both analyses of the ITS2 and COI sequences. There were positive values of Tajima's D in both ITS2 and COI analyses indicating a decreasing population size or balancing selection and a low level of both low and high-frequency polymorphisms [29].

There were negative values of Fu and Li's F and D in both analyses of the ITS2 and COI sequences from all countries in this study. An excess of singletons and a high number of unshared haplotypes were indicated by their negative values of it [30]. The deficiency of alleles from a recent population bottleneck can be due to a genetic drift causing reducing population size as indicated by positive values of Fu's Fs statistic [31].

According to the pairwise F_{ST} values, a huge genetic differentiation between populations was observed from the high F_{ST} values of Iran-Cambodia, Iran-China, and Iran-Sri Lanka in the ITS2 analysis and Iran-Sri Lanka, and Iran-Pakistan in the COI analysis. A high genetic differentiation might be achieved due to the large geographical distance between the two locations [27]. When the distance between the populations increases, the genetic similarity between the populations was decreased. That suggests the isolation by distance that is derived from spatially limited gene flow [32]. Zero F_{ST} values were obtained for Cambodia-China, Cambodia-Sri Lanka and China-Sri Lanka in the ITS2 analysis and a negative F_{ST} value was obtained for Sri Lanka-Pakistan in the

COI analysis. F_{ST} value is close to zero when the allele frequencies of two subpopulations are very similar and not differentiated [33]. High rates of gene flow and the absence of genetic subdivision between the considered populations were indicated by low F_{ST} values.

Anopheles fossil record is very uncommon as there were only two *Anopheles* fossils recognized currently named *Anopheles* (*Nyssorhynchus*) *dominicanus* from the Late Eocene (33.9–41.3 Mya) being the oldest one and *Anopheles rottensis* from the Late Oligocene (13.8–33.9 Mya) being the most recent one [22]. The usage of those records seemed to be problematic based on the applied dating technique [34]. Therefore, the diversification date of the most recent common ancestor (MRCA) of the *Anopheles* genus was used to calibrate the phylogeographic trees in the study. The diversification of the *Anopheles* genus was dated in this study ranging from 81.15 to 85.09 Mya and 80.93 to 85.07 Mya based on ITS2 and COI phylogeographic analysis respectively in the Cretaceous.

The link between the diversification of anophelines and malaria parasites is still controversial [35]. The origin of *P. falciparum* was dated at 2.5 Mya and the initial radiation of mammalian *Plasmodium* at 12.8 Mya [36]. The divergence time of these species was younger than which inferred for their *Anopheles* vectors in our analysis.

The diversification of *An. culicifacies* species complex was obtained as ranging from 20.25 to 24.12 Mya and 22.37 to 26.22 Mya based on ITS2 and COI phylogeographic analysis respectively. That was nearly equal to the divergence of *An. culicifacies* based on mitochondrial genome PCG123 datasets were obtained as 21.76 Mya [37]. The diversification of the clade 2 including the sibling species B, C and E was earlier than the clade 1 with sibling species A and D as shown in the ITS2 phylogeographic analysis. The divergence of the clade 1 and 2 occurred prior to

splitting into the sibling species. The diversification of sibling species of the species complex mainly occurred in the Miocene.

The split time of *An. subpictus* was more recent than the split time of *An. culicifacies* in this study that were obtained as 12.2 Mya and 9.25 Mya in ITS2 and COI phylogeographic analysis respectively. When considering other species of the genus *Anopheles*, the diversification of *Anopheles gambiae* / South East Asia-Oceania anophelines, *Anopheles darlingi* / *Anopheles albitarsis* complex and *Anopheles aquasalis* / *Anopheles albitarsis* complex was dated at 65.5 Mya, 38.98 Mya and 28.56 Mya respectively [24]. Therefore, the diversification of *An. culicifacies* species complex obtained in this study was more recent than these species of the *Anophele*s genus.

According to the obtained dates of the phylogeographic analysis, the sibling species B was considered as the earliest evolved sibling species based on ITS2 sequences and when considering COI sequences it was the sibling species A. The recently evolved sibling species were C and D respectively in ITS2 and COI analysis. Based on previous studies, it was mentioned that initially the sibling species A and B evolved from a common ancestor and the sibling species D diverged from A and the sibling species C from the sibling species B later [9]. The diversification times of the sibling species were not mentioned in previous studies.

The diversification dates of the earliest members of the sibling species varied between ITS2 and COI analysis which can be due to the changes in the diversification of the nuclear genome and the mitochondrial genome. It is assumed that the mtDNA evolves at a faster rate than nuclear DNA (nuDNA) in animals and the ratio of mtDNA over nuDNA mutation rate varies between 2 and 6 in invertebrates including insects and arachnids [38].

Most of the intraspecific divergence dates to the Pliocene in the study. In ITS2 phylogeographic analysis, intraspecies diversification was observed mostly in the Pliocene period in India, Iran, Cambodia, Sri Lanka and China as well as in COI analysis it was observed in countries of Pakistan, India, Sri Lanka and Cambodia. The climate was warm and humid during this period and tropical forest is likely to have been widespread across Southeast Asia and larval habitats abundant as well as the forest fragmentation is also increased. Therefore, it can be increased the niche availability and facilitated the spread [39]. All Pleistocene-dated divergence events can be observed in the COI phylogeographic analysis in the countries of Iran, India, Sri Lanka, Oman and the United Arab Emirates. It was indicating that the Pleistocene glaciations have shaped genetic diversity within species and possibly to have retained sacks of forest habitat at intermediate elevations throughout the Pleistocene [39].

The possibility of *An. culicifacies* species complex maintaining a relatively high effective population size despite of the enormous population fluctuations was suggested. Therefore, it is necessary to revisit the mosquito population to achieve success from the vector control strategies while incorporating the information on the genetic markers including both mitochondrial and nuclear markers to enhance the robustness of the reported observations.

Conclusions

The importance of understanding the genetic diversity, phylogenetic and phylogeographic relationships of mosquito vectors using ITS2 and COI sequences of *An. culicifacies* species complex was focused. The sibling species of *An. culicifacies* species complex was broadly divided into two clades as A, D and B, C, and E in both phylogenetic and phylogeographic analysis using ITS2 sequences. There were many clades in both phylogenetic and phylogeographic analysis using

COI sequences due to the highly divergent COI sequences and the higher mutation rate of the mitochondrial genome than the nuclear genome. There was more genetic divergence with less selective pressure and a recent diversification shown in the sibling species A and D than the sibling species B, C and E from their MRCA. The high genetic diversity of *An. culicifacies* species complex suggests the likelihood of maintaining a relatively high effective population size despite the vector control efforts. The genetic diversities of vectors of mosquito-borne diseases should be considered in implementing effective vector control strategies as uniform control measures may not be equally effective for all the populations of a single species.

Supporting information

S1 Table. GenBank accession number, country of origin, sibling species of ITS2 sequences of *An. culicifacies*.S2 Table. Region and country of origin of *Aedes aegypti* Cytochrome C Oxidase 1 (CO1). (S1_Table.pdf)

S1 Table. GenBank accession number, country of origin, sibling species of COI sequences of *An. culicifacies*. (S2_Table.pdf)

S2 Table. GenBank accession numbers of ITS2 and COI sequences of the species used as outgroup of phylogenetic trees and phylogeographic trees. (S3_Table.pdf)

S4 Table. GenBank accession numbers of the sequences included in clade 1, 2 and outgroup of Bayesian likelihood tree generated by MrBayes-3.2.5_WIN32_x86 software using ITS2 sequences of *An. culicifacies*. (S4_Table.pdf)

S5 Table. GenBank accession numbers of the sequences included in Clade 1 to 4 and outgroup of Bayesian likelihood tree generated by MrBayes-3.2.5_WIN32_x86 software using COI sequences of *An. culicifacies*. (S5_Table.pdf)

S6 Table. Haplotype frequencies and haplotype diversities (Hd) in populations of ITS2 haplotype network of *An. culicifacies* species complex obtained using Arlequin version 3.5.2.2 and DnaSP v5 software. (S6_Table.pdf)

S7 Table. Haplotype frequencies and haplotype diversities (Hd) in populations of COI haplotype network of *An. culicifacies* species complex obtained using Arlequin version 3.5.2.2 and DnaSP v5 software. (S7_Table.pdf)

S8 Table. GenBank accession numbers of ITS2 sequences included in clade 1, 2 and outgroup of phylogeographic tree generated by BEAST v1.8.2 software using ITS2 sequences of *An. culicifacies.* (S8_Table.pdf)

S9 Table. GenBank accession numbers of COI sequences included in clade 1 to 3 and outgroup of phylogeographic tree generated by BEAST v1.8.2 software using COI sequences of An. *culicifacies.* (S9_Table.pdf)

Acknowledgments

All the members of the Genetics and Molecular Biology Unit at the University of Sri Jayewardenepura, Sri Lanka were greatly acknowledged for the assistance rendered during this study.

References

[1] Surendran SN, Abhayawardana TA, De Silva BGDNK, Ramasamy R, Ramasamy MS. *Anopheles culicifacies* Y-chromosome dimorphism indicates sibling species (b and e) with different malaria vector potential in Sri Lanka. Med. Vet. Entomol. 2000; 14(4): 437–440. doi: 10.1046/j.1365-2915.2000.00264.x.

[2] Shanika KHS, Harischandra IN, De Silva BGDNK. Nucleotide sequence and secondary structure variations in ITS2-rDNA region of the members of *Anopheles culicifacies* (Diptera : Culicidae) species complex Nucleotide Sequence and Secondary Structure Variations in ITS2-rDNA Region of the Members of *Anopheles culicifacies* (Diptera: Culicidae) Species Complex. Vidyodaya J. of Sci. 2016; 20: 26-40.

[3] Sharma VP, Dev V. Biology & control of *Anopheles culicifacies* Giles 1901. Indian J. Med. Res. 2015; 142: 525–536.

[4] Goswami G, Raghavendra K, Nanda N, Gakhar SK, Subbarao SK. PCR-RFLP of mitochondrial cytochrome oxidase subunit II and ITS2 of ribosomal DNA: Markers for the identification of members of the *Anopheles culicifacies* complex (Diptera: Culicidae). Acta Trop. 2005; 95(2): 92–99. doi: 10.1016/j.actatropica.2005.04.018.

[5] Van Bortel W, Sochanta T, Harbach RE, Socheat D, Roelants P, Backeljau T, et al. Presence of *Anopheles culicifacies* B in Cambodia established by the PCR-RFLP assay developed for the identification of *Anopheles minimus* species A and C and four related species. Med. Vet. Entomol. 2002; 16(3): 329–334. doi: 10.1046/j.1365-2915.2002.00376.x.

[6] Surendran SN, Hawkes NJ, Steven A, Hemingway J, Ramasamy R. Molecular studies of *Anopheles culicifacies* (Diptera: Culicidae) in Sri Lanka: Sibling species B and E show

sequence identity at multiple loci. Eur. J. Entomol. 2006; 103(1): 233–237. doi: 10.14411/eje.2006.025.

[7] Kar I, Subbarao SK, Eapen A, Ravindran J, Satyanarayana TS, Raghavendra K, et al. Evidence for a new malaria vector species, species E, within the *Anopheles culicifacies* complex (Diptera: Culicidae). Journal of medical entomology. 1999; 36(5): 595-600.

[8] Dassanayake RS, Gunawardene YINS, De Silva BGDNK. ITS-2 secondary structures and phylogeny of *Anopheles culicifacies* species. Bioinformation. 2008; 2(10): 456–460. doi: 10.6026/97320630002456.

[9] Surendran SN, Ramasamy R. The *Anopheles culicifacies* and *An. subpictus* species complexes in Sri Lanka and their implications for Malaria control in the country. Trop. Med. Health. 2010; 38(1): 1–11. doi: 10.2149/tmh.2009-12.

[10] Tyagi V, Sharma AK, Dhiman S, Srivastava AR, Yadav R, Sukumaran D, et al. Malaria vector *Anopheles culicifacies* sibling species differentiation using egg morphometry and morphology. Parasites & Vectors. 2016; 9(1): 1-13. https://doi.org/10.1186/s13071-016-1478-5.

[11] Prasad A, Mathur P, Srivastava M, Sharma E, Parveen A, Kumar D. Ecology and Behaviour of *Anopheles culicifacies* sensu lato (s.l.). A General review. International Journal of Current and Academic Review. 2015; 3(5): 227-241.

[12] Subbarao SK, Vasantha K, Raghavendra K, Sharma VP, Sharma GK. *Anopheles culicifacies*: sibling species composition and its relationship to malaria incidence. Journal of American Mosquito Control Association. 1988; 4(1): 29-33.

[13] Surendran SN, Hawkes NJ, Andrew S, Hemingway J,Ramasamy R. Molecular studies of *Anopheles culicifacies* (Diptera: Culicidae) in Sri Lanka: sibling species B and E show sequence identity at multiple loci. European Journal of Entomology. 2006; 103(1): 233–237.

[14] Hu Y, Fan H, Chen Y, Chang J, Zhan X, Wu H, et al. Spatial patterns and conservation of genetic and phylogenetic diversity of wildlife in China. Science Advances. 2021; 7(4): p.eabd5725.

[15] Avise JC. Phylogeography: the history and formation of species. Harvard university press. 2000

[16] Rius M, Turon X. Phylogeography and the description of geographic patterns in invasion genomics. Frontiers in Ecology and Evolution. 2020; 8: p.595711.

[17] Lorenz C, Alves JM, Foster PG, Suesdek L, Sallum MAM. Phylogeny and temporal diversification of mosquitoes (Diptera: Culicidae) with an emphasis on the Neotropical fauna. Systematic Entomology. 2021; 46(4): 798-811.

[18] Karimian F, Oshaghi MA, Sedaghat MM, Waterhouse RM, Vatandoost H, Hanafi-Bojd AA, et al. Phylogenetic analysis of the oriental-palearctic-afrotropical members of *Anopheles* (Culicidae: Diptera) based on nuclear rDNA and mitochondrial DNA characteristics. Jpn. J. Infect. Dis. 2014; 67(5): 361–367. doi: 10.7883/yoken.67.361.

[19] Depaquit J, Lienard E, Verzeaux-Griffon A, Ferte H, Bounamous A, Gantier J, et al. Molecular homogeneity in diverse geographical populations of Phlebotomus papatasi (Diptera, Psychodidae) inferred from ND4 mtDNA and ITS2 rDNA. Epidemiological consequences. Infect. Genet. 2008; Evol. 8(2): 159–170, 2008, doi: 10.1016/j.meegid.2007.12.001.

[20] Jayatunga DPW, Harischandra IN, Chandrasekharan NV, de Silva BGDNK. Compensatory base changes reveal sexual incompatibility among members of the *Anopheles subpictus* sensu lato (Diptera: Culicidae) species complex in Sri Lanka. Life. 2021; 11(3). doi: 10.3390/life11030211.

[21] Rangel-Gamboa L, Martínez-Hernandez F, Maravilla P, Arenas-Guzmán R, Flisser A. Update of phylogenetic and genetic diversity of Sporothrix schenckii sensu lato. Med. Mycol. 2016; 54(3): 248–255. doi: 10.1093/mmy/myv096.

[22] Freitas LA, Russo CAM, Voloch CM, Mutaquiha OCF, Marques LP, Schrago CG. Diversification of the genus *Anopheles* and a neotropical clade from the Late Cretaceous. PLoS One. 2015; 10(8): 1–12. doi: 10.1371/journal.pone.0134462.

[23] Lafontaine DLJ, Tollervey D. The function and synthesis of ribosomes. Nat. Rev. Mol. Cell Biol. 2001; 2(7): 514–520. doi: 10.1038/35080045.

[24] Martinez-Villegas L, Assis-Geraldo J, Koerich LB, Collier TC, Lee Y, Main BJ, et al. Characterization of the complete mitogenome of Anopheles aquasalis, and phylogenetic divergences among Anopheles from diverse geographic zones. PLoS One. 2019; 14(9): 1–22. doi: 10.1371/journal.pone.0219523.

[25] Gaunt MW, Miles MA. An insect molecular clock dates the origin of the insects and accords with palaeontological and biogeographic landmarks. Mol. Biol. Evol. 2002; 19(5): 748–761. doi: 10.1093/oxfordjournals.molbev.a004133.

[26] Weeraratne TC, Surendran SN, Reimer LJ, Wondji CS, Perera MDB, Walton C, et al. Molecular characterization of Anopheline (Diptera: Culicidae) mosquitoes from eight geographical locations of Sri Lanka. Malar. J. 2017; 16(1): 1–14. doi: 10.1186/s12936-017-

1876-y.

[27] Mayoke A, Muya SM, Bateta R, Mireji PO, Okoth SO, Onyoyo SG, et al. Genetic diversity and phylogenetic relationships of tsetse flies of the palpalis group in Congo Brazzaville based on mitochondrial cox1 gene sequences. Parasites and Vectors. 2020; 13(1): 1–16. doi: 10.1186/s13071-020-04120-3.

[28] Dharmarathne HAKM, Weerasena OVDSJ, Perera KLNS, Galhena G. Genetic characterization of *Aedes aegypti* (Diptera: Culicidae) in Sri Lanka based on COI gene. J. Vector Borne Dis. 2020; 57(2): 153–160. doi: 10.4103/0972-9062.310871.

[29] Panhuis TM, Swanson WJ. Molecular evolution and population genetic analysis of candidate female reproductive genes in *Drosophila*. Genetics. 2006; 173(4): 2039–2047. doi: 10.1534/genetics.105.053611.

[30] Fernando HSD, Hapugoda M, Perera R, Black WC, de Silva BGDNK. Mitochondrial metabolic genes provide phylogeographic relationships of global collections of *Aedes aegypti* (Diptera: Culicidae). PLoS One. 2020; 15(7): 1–18. doi: 10.1371/journal.pone.0235430.

[31] Sharma M, Fomda BA, Mazta S, Sehgal R, Singh BB, Malla N. Genetic diversity and population genetic structure analysis of *Echinococcus granulosus* sensu stricto complex based on mitochondrial DNA signature. PLoS One. 2013; 8(12): 1–8. doi: 10.1371/journal.pone.0082904.

[32] Jensen JL, Bohonak AJ, Kelley ST. Isolation by distance, web service. BMC Genet. 2005; 6: 1–6. doi: 10.1186/1471-2156-6-13.

[33] Jacquot M. Genomic diversity of pathogenic bacteria in the *Borrelia burgdorferi* species complex: evolution and molecular epidemiology. PhD thesis. Blaise Pascal University. 2014.

[34] Logue K, Chan ER, Phipps T, Small ST, Reimer L, Henry-Halldin C, Sattabongkot J, et al. Mitochondrial genome sequences reveal deep divergences among *Anopheles punctulatus* sibling species in Papua New Guinea. Malar. J. 2013; 12(1): 1–11. doi: 10.1186/1475-2875-12-64.

[35] Moreno M, Marinotti O, Krzywinski J, Tadei WP, James AA, Achee NL, et al. Complete mtDNA genomes of *Anopheles darlingi* and an approach to anopheline divergence time. Malar. J. 2010; 9(1): 1–13. doi: 10.1186/1475-2875-9-127.

[36] Ricklefs RE, Outlaw DC. A molecular clock for malaria parasites. Science. 2010; 329(5988): 226–229. doi: 10.1126/science.1188954.

[37] Hao YJ, Zou YL, Ding YR, Xu WY, Yan ZT, Li XD, et al. Complete mitochondrial genomes of *Anopheles stephensi* and *An. dirus* and comparative evolutionary mitochondriomics of 50 mosquitoes. Sci. Rep. 2017; 7(1): 1–13. doi: 10.1038/s41598-017-07977-0.

[38] Allio R, Donega S, Galtier N, Nabholz B. Large variation in the ratio of mitochondrial to nuclear mutation rate across animals: Implications for genetic diversity and the use of mitochondrial DNA as a molecular marker. Mol. Biol. Evol. 2017; 34(11): 2762–2772. doi: 10.1093/molbev/msx197.

[39] Morgan K, O'Loughlin SM, Mun-Yik F, Linton YM, Somboon P, Min S, et al. Molecular phylogenetics and biogeography of the Neocellia Series of *Anopheles* mosquitoes in the

Oriental Region. Molecular Phylogenetics and Evolution. 2009; 52(3): 588-601.

Figure

Click here to access/download;Figure;Fig 1.tif

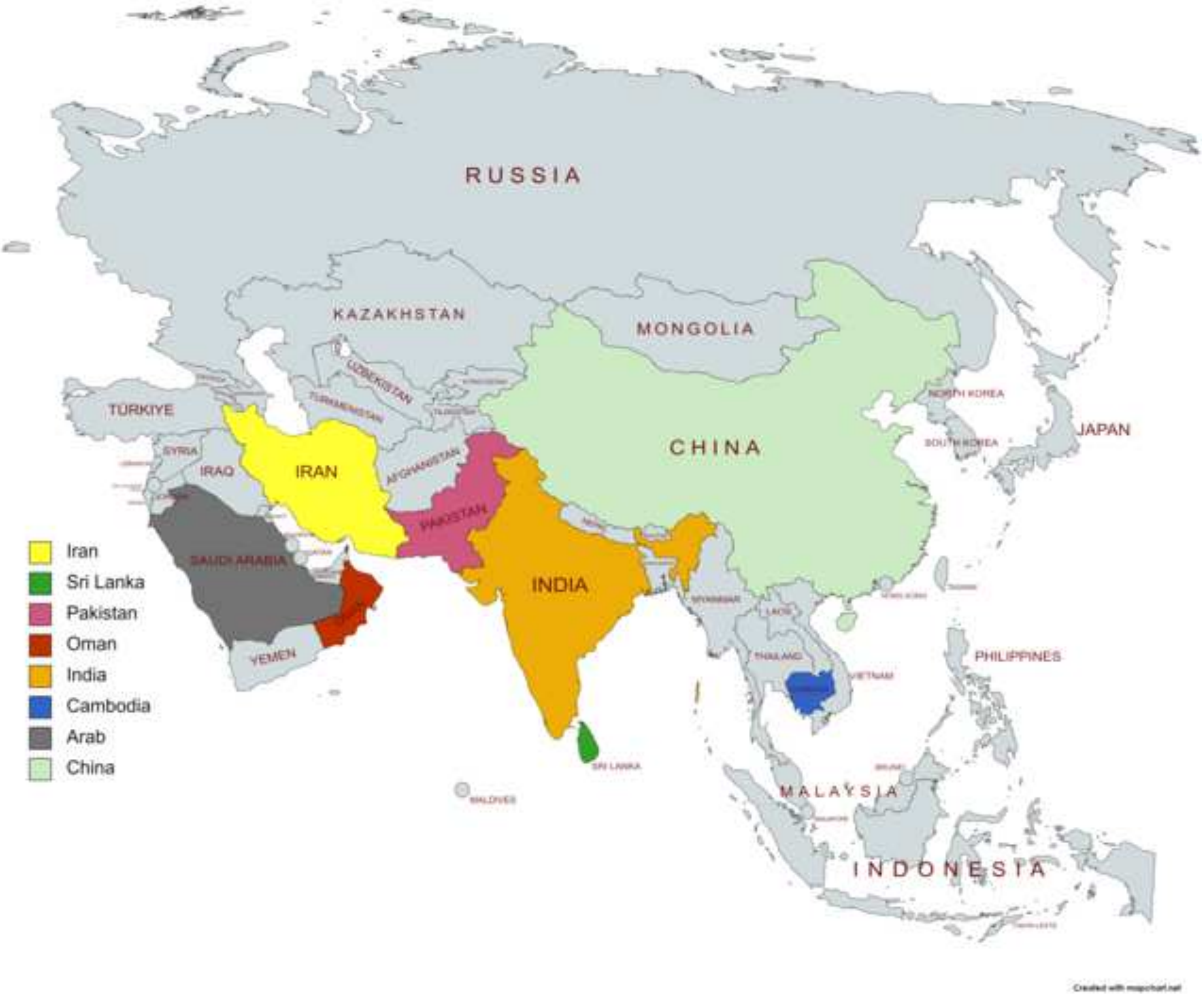

Figure
Click here to access/download;Figure;Fig 2.tif

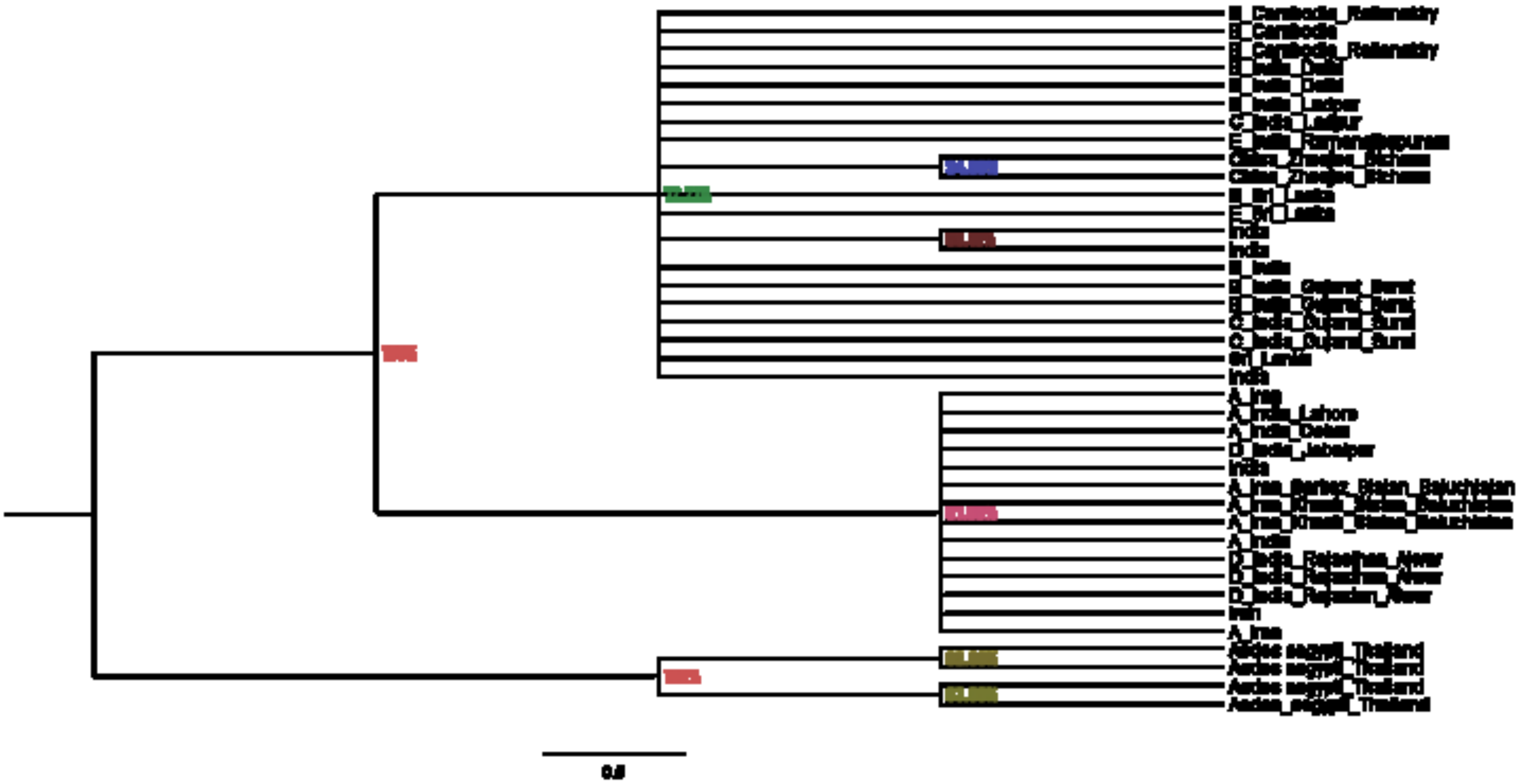

Figure Click here to access/download;Figure;Fig 3.tif

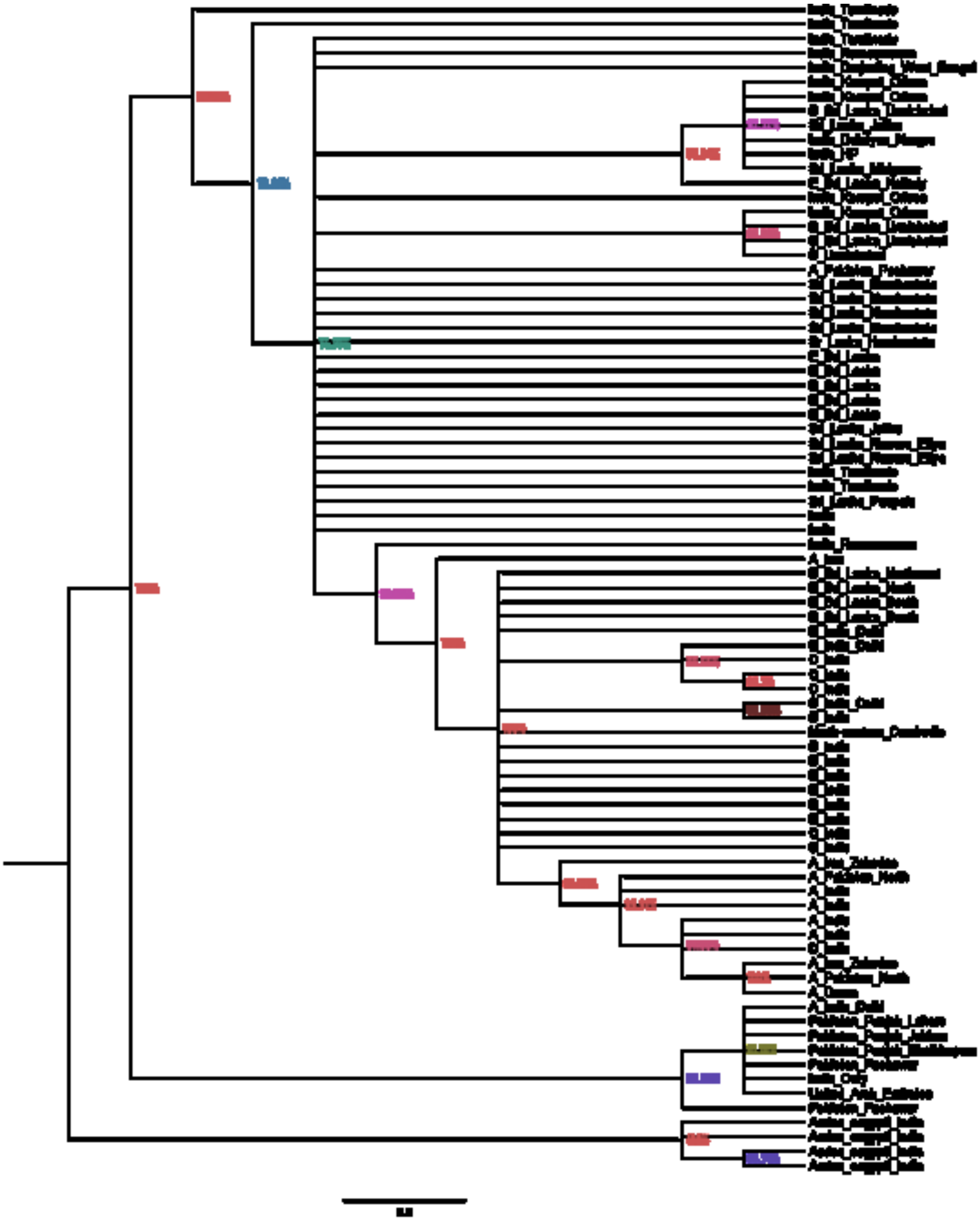

Figure

Click here to access/download;Figure;Fig 4.tif

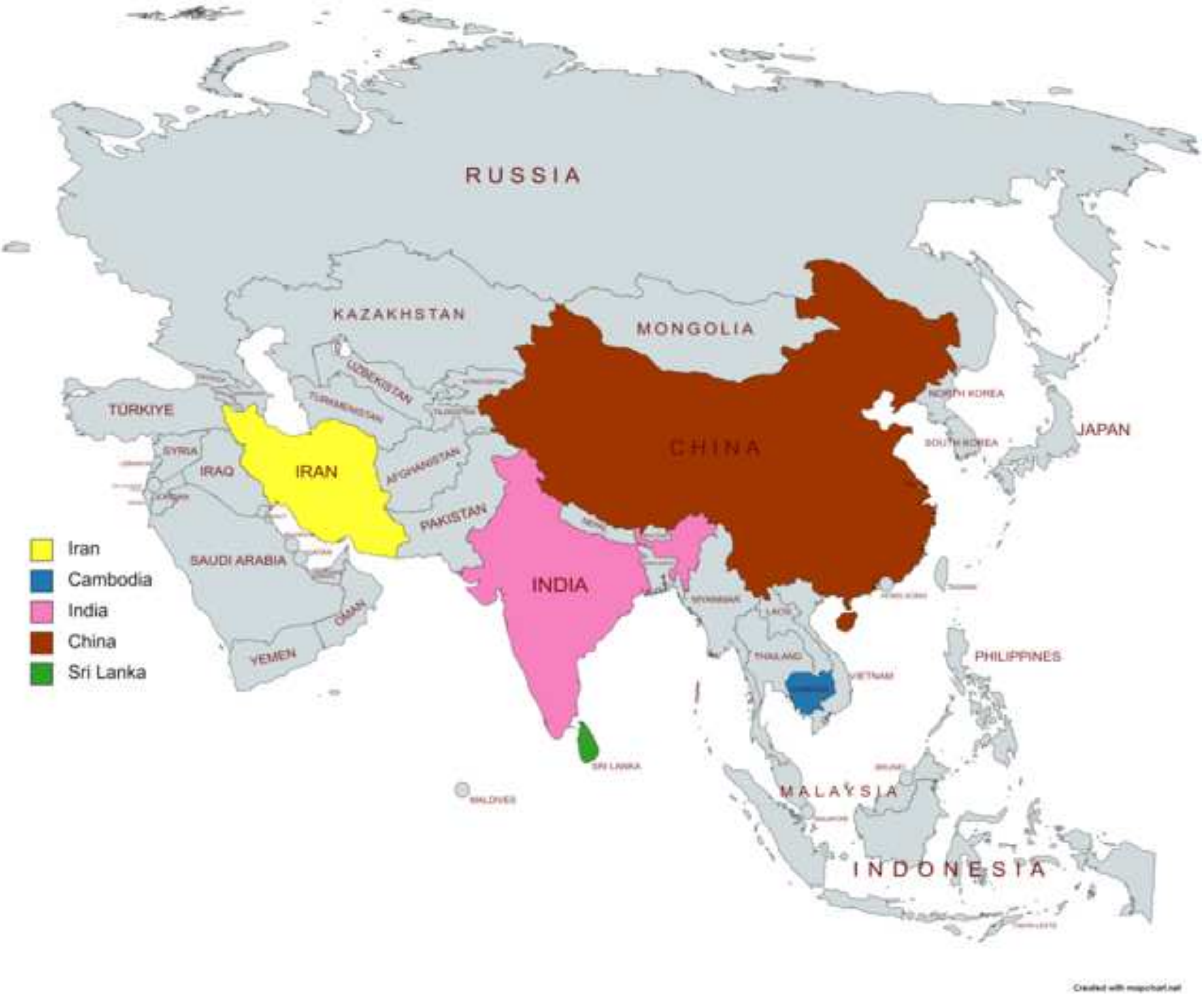

Figure

Click here to access/download;Figure;Fig 5.tif

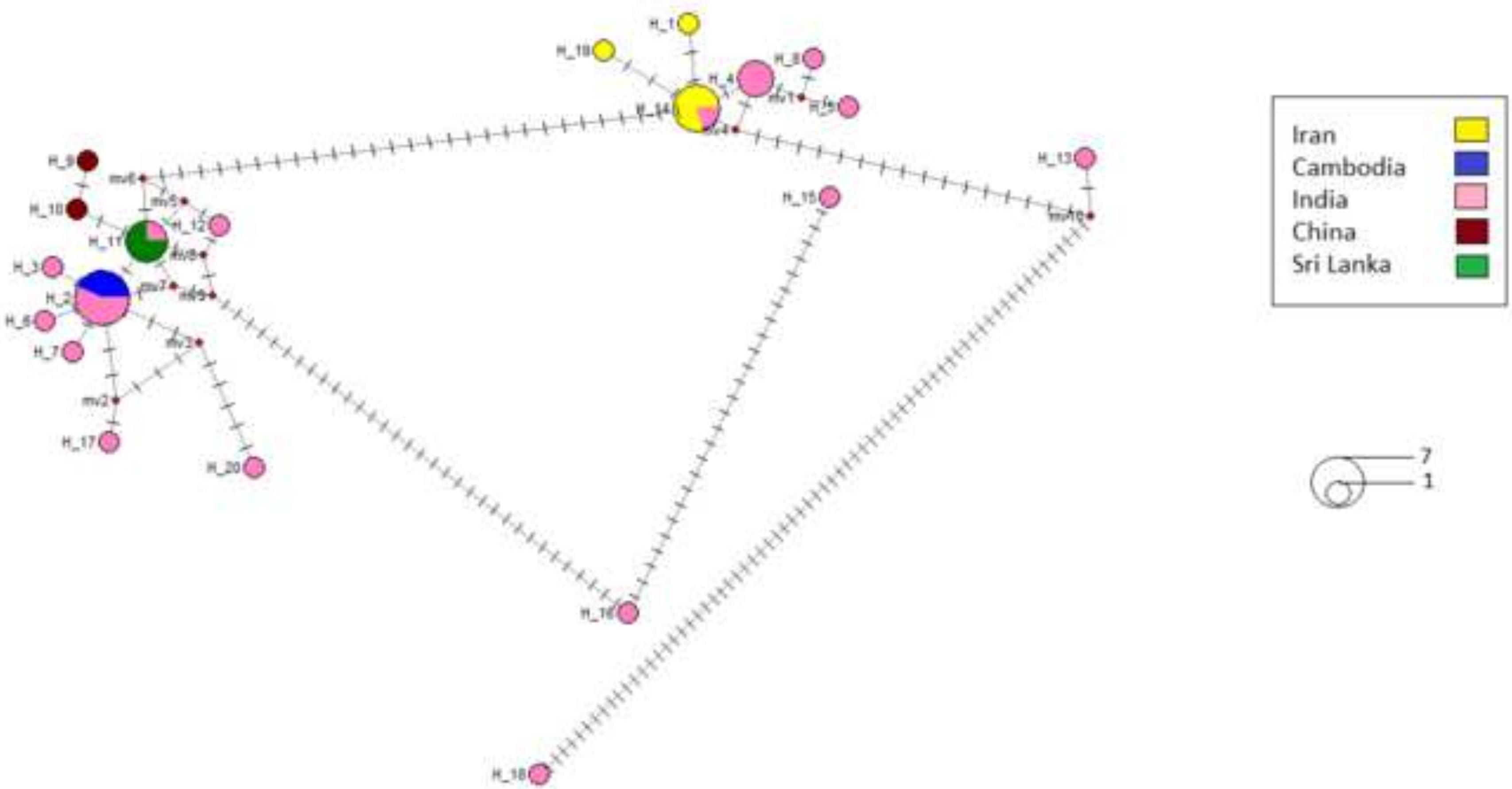

Figure

Click here to access/download;Figure;Fig 6.tif

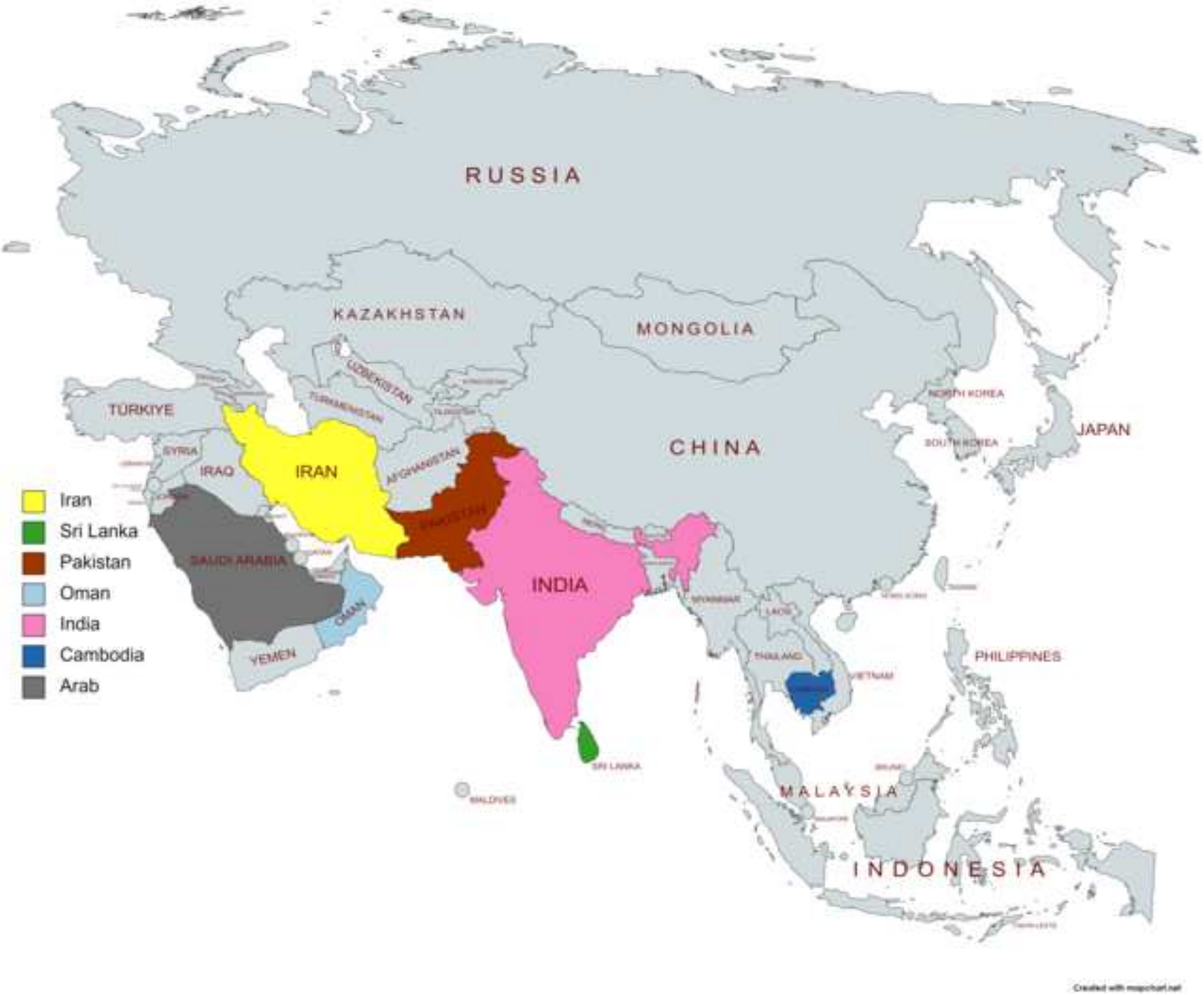

Figure

Click here to access/download;Figure;Fig 7.tif

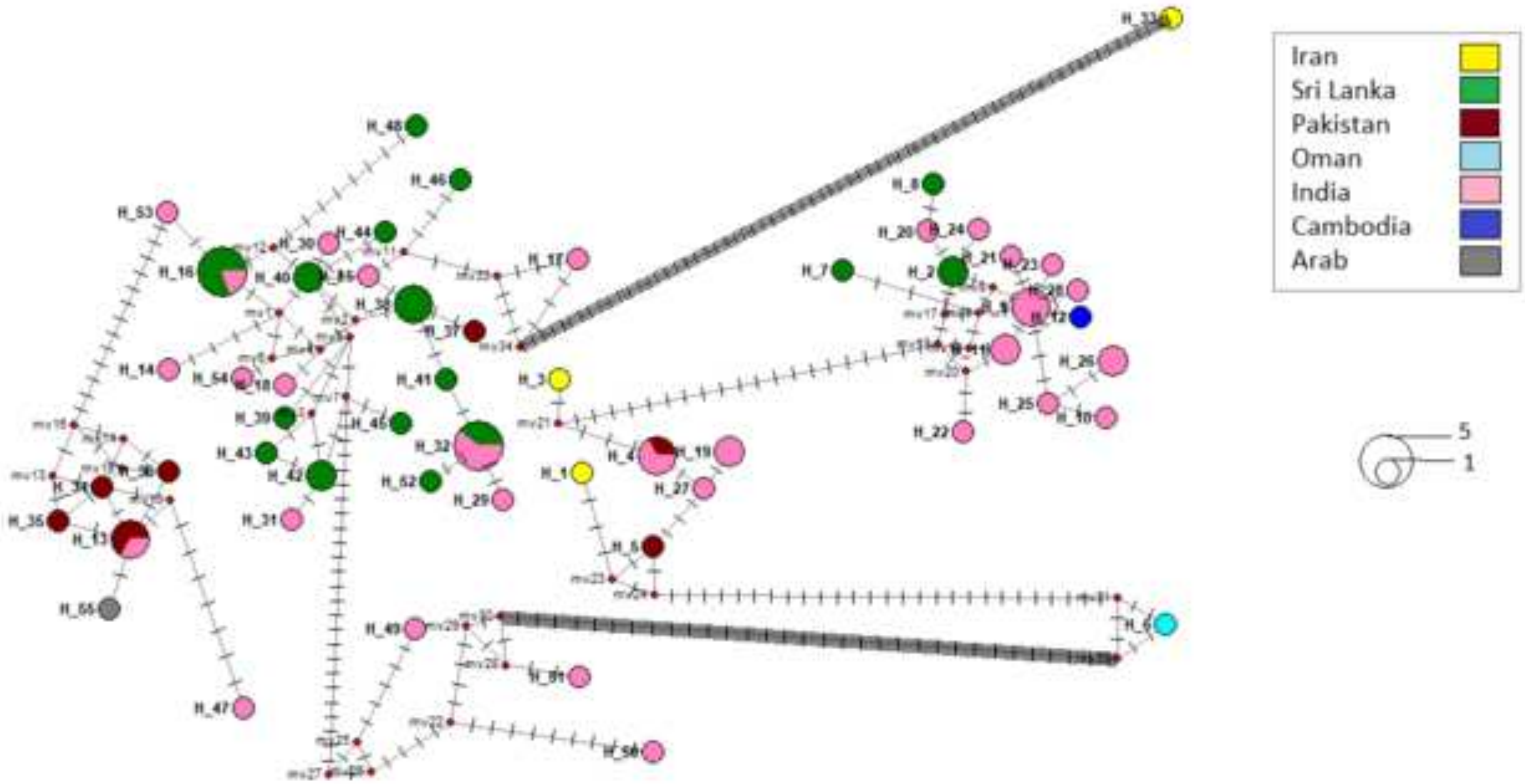

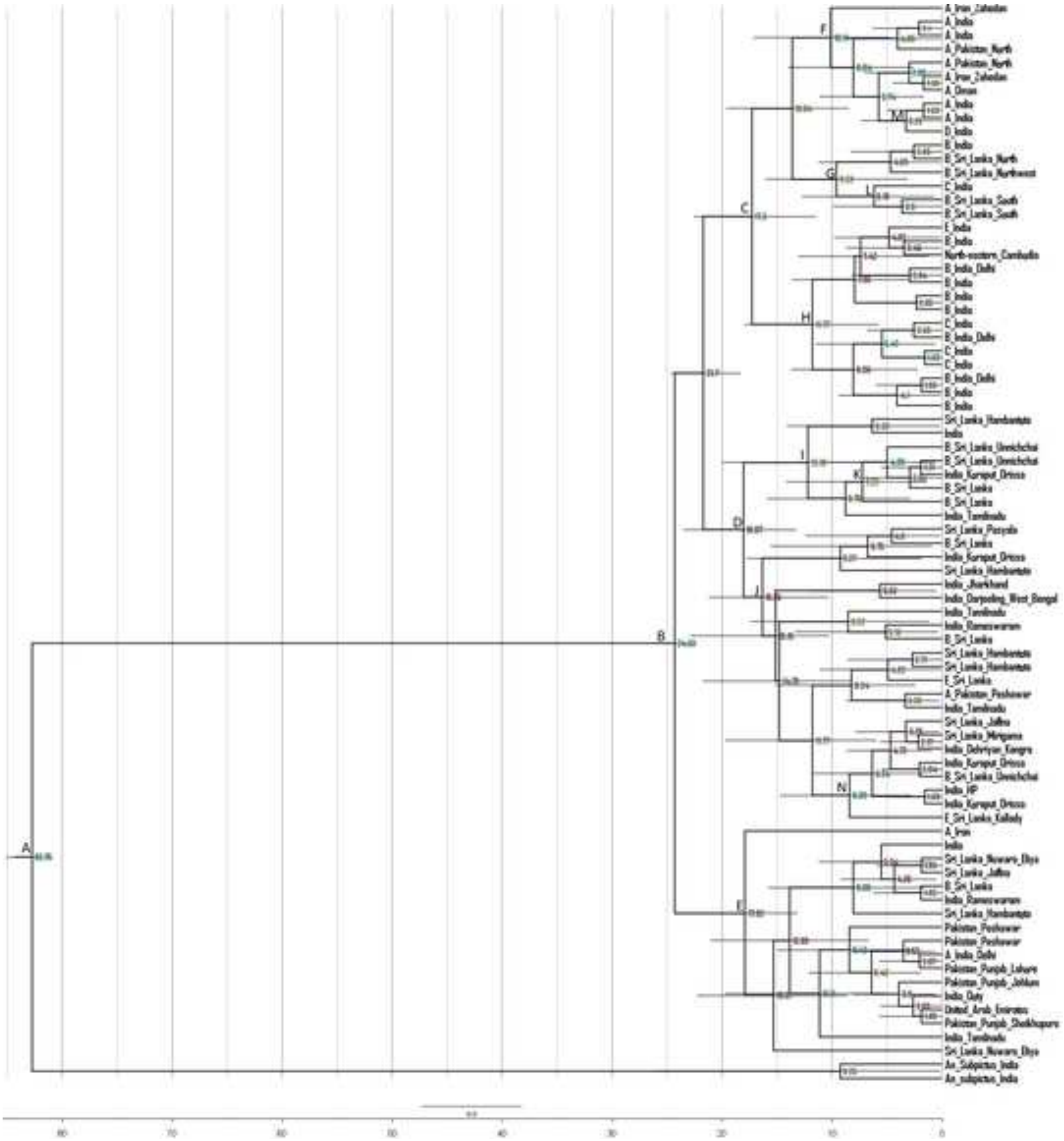
A
B
C
D
E
F
G
H
I
J
K
L
M
N
A_Iran_Zahedan
A_India
A_India
A_Pakistan_North
A_Pakistan_North
A_Iran_Zahedan
A_Oman
A_India
A_India
D_India
B_India
B_Sri_Lanka_North
B_Sri_Lanka_Northwest
C_India
B_Sri_Lanka_South
B_Sri_Lanka_South
E_India
B_India
North-eastern_Cambodia
B_India_Delhi
B_India
B_India
B_India
C_India
B_India_Delhi
C_India
C_India
B_India_Delhi
B_India
B_India
Sri_Lanka_Hambantota
India
B_Sri_Lanka_Unnichchai
B_Sri_Lanka_Unnichchai
India_Koraput_Orissa
B_Sri_Lanka
B_Sri_Lanka
India_Tamilnadu
Sri_Lanka_Pasyala
B_Sri_Lanka
India_Koraput_Orissa
Sri_Lanka_Hambantota
India_Jharkhand
India_Darjeeling_West_Bengal
India_Tamilnadu
India_Rameswaram
B_Sri_Lanka
Sri_Lanka_Hambantota
Sri_Lanka_Hambantota
E_Sri_Lanka
A_Pakistan_Peshawar
India_Tamilnadu
Sri_Lanka_Jaffna
Sri_Lanka_Mirigama
India_Dehriyan_Kangra
India_Koraput_Orissa
B_Sri_Lanka_Unnichchai
India_HP
India_Koraput_Orissa
E_Sri_Lanka_Kallady
A_Iran
India
Sri_Lanka_Nuwara_Eliya
Sri_Lanka_Jaffna
B_Sri_Lanka
India_Rameswaram
Sri_Lanka_Hambantota
Pakistan_Peshawar
Pakistan_Peshawar
A_India_Delhi
Pakistan_Punjab_Lahore
Pakistan_Punjab_Jehlum
India_Ooty
United_Arab_Emirates
Pakistan_Punjab_Sheikhupura
India_Tamilnadu
Sri_Lanka_Nuwara_Eliya
An_Subpictus_India
An_subpictus_India
80
70
60
50
40
30
20
10
0

Figure

Click here to access/download;Figure;Fig 9.tif

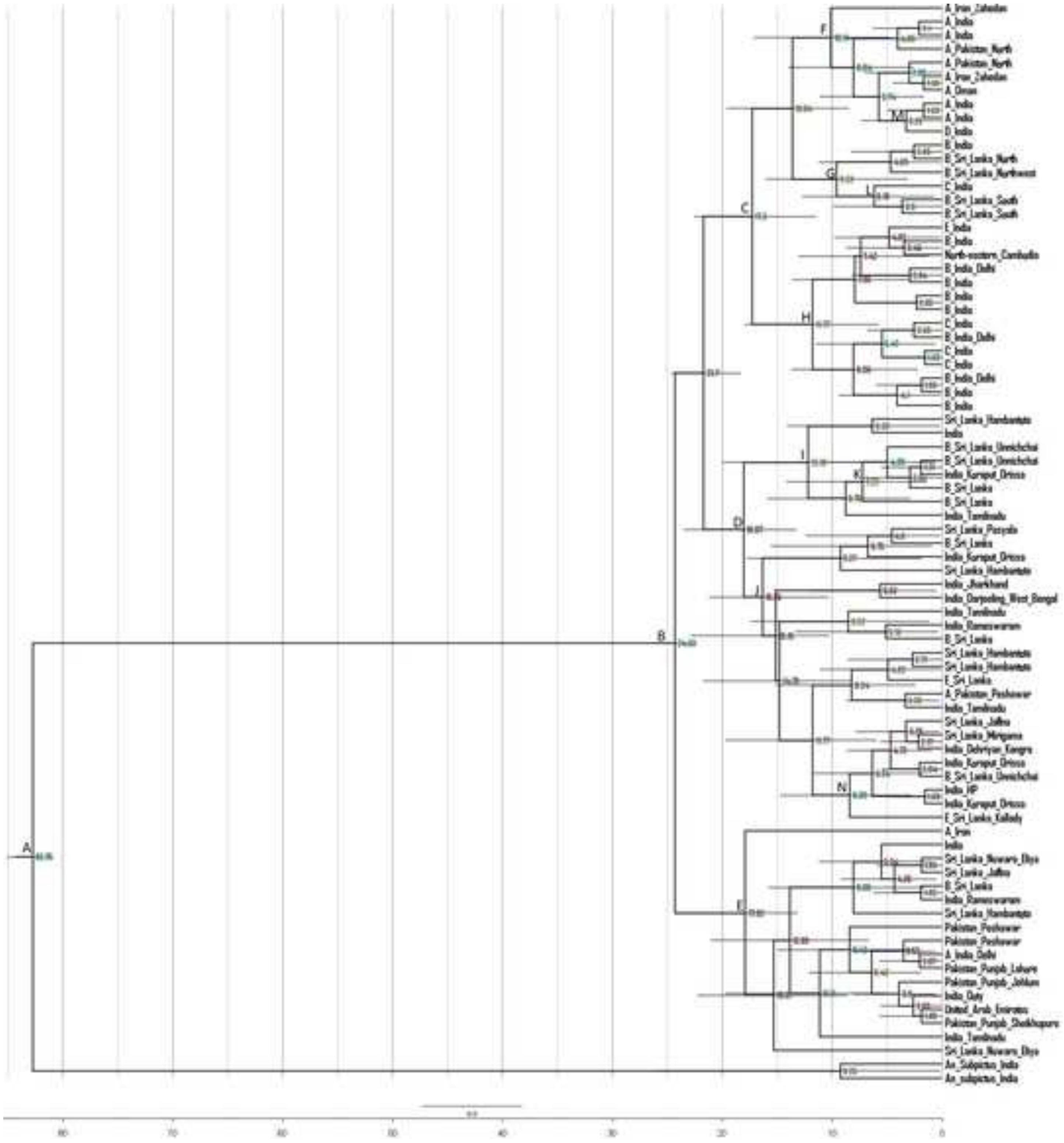

Click here to access/download
Supporting Information
S1_Table.pdf

Click here to access/download
Supporting Information
S2_Table.pdf

Click here to access/download
Supporting Information
S3_Table.pdf

Click here to access/download
Supporting Information
S4_Table.pdf

Click here to access/download
Supporting Information
S5_Table.pdf

Click here to access/download
Supporting Information
S6_Table.pdf

Click here to access/download
Supporting Information
S7_Table.pdf

Click here to access/download
Supporting Information
S8_Table.pdf

Click here to access/download
Supporting Information
S9_Table.pdf
