# Supplementary material for: Genetic diversity, phylogenetic and phylogeographic analysis of Anopheles culicifacies species complex using ITS2 and COI sequences
Source: PLoS One. 2023 Aug 16;18(8):e0290178. doi: 10.1371/journal.pone.0290178 (PMC10431676; doi:10.1371/journal.pone.0290178)
Supplement: Attachment — Submitted filename: Reviewer comments_PLOS ONE.doc [file pone.0290178.s014.doc]

Dear Editor,

I have reviewed the manuscript PONE-D-23-08529, entitled “Genetic diversity, phylogenetic and phylogeographic analysis of *Anopheles culicifacies* species complex using ITS2 and COI sequences” submitted to PLOS ONE as a full-length article. The article is well written and the content is worth to be published. In my opinion this paper can be accepted for publication in PLOS ONE after minor revision.

Kind regards,

Ranju Manoj

_____________________________

Ranju Manoj, DVM, MVSc., PhD

Post-doctoral associate and resident in Veterinary Parasitology,

Department of Population Medicine and Diagnostics,

AHDC, Cornell University,

240, Farrier Road, Ithaca, New York-14850.

Tel +1 6465207636

e.mail: rm846@cornell.edu

drranjuvet@gmail.com

**COMMENTS TO THE AUTHORS**

The manuscript PONE-D-23-08529, entitled “Genetic diversity, phylogenetic and phylogeographic analysis of *Anopheles culicifacies* species complex using ITS2 and COI sequences” is a molecular data analyses to infer the phylogenetic and phylogeographic relationships of *An. culicifacies* species complex targeting ITS2 and COI sequences available in the GenBank.

Here in this article, authors are assessing the phylogenetic and phylogeographic relationships between the sibling species of the *An. culicifacies* species complex while observing their genetic diversity and genetic differentiation. In general, the article is well written except for some repetition of results in the discussion section. I have minor suggestions, which have been mentioned in the attached pdf.
